# Simple Strategies to Quantify and Control Polymer Threading into Micropores

**DOI:** 10.1021/jacs.5c07556

**Published:** 2025-06-09

**Authors:** Supreet Kaur, Benjamin Lesea-Pringle, Surya Marjit, Hyukhun Hong, Aqib Rahman, Boran Ma, Denize C. Favaro, Christopher DelRe

**Affiliations:** † Nanoscience Initiative, 14772 CUNY Advanced Science Research Center, New York, New York 10031, USA; ‡ Ph.D. Program in Biochemistry, The Graduate Center of the City University of New York, 365 fifth Ave, New York, New York 10016, USA; § Department of Chemistry and Chemical Biology, 1812 Harvard University, Cambridge, Massachusetts 02138, USA; ∥ School of Polymer Science and Engineering, 5104 University of Southern Mississippi, 118 College Drive, Hattiesburg, Mississippi 39406, USA; ⊥ Structural Biology Initiative, CUNY Advanced Science Research Center, New York, New York 10031, USA; # Department of Chemistry and Biochemistry, City College of New York, 275 Convent Ave, New York, New York 10031, USA

## Abstract

Blends of polymers with microporous particles are essential to many modern technologies, including membranes, catalysts, nanocomposites, and porous liquids. However, the design space and performance of these technologies are substantially limited because it is difficult to quantify and control how polymers thread into the particles’ sub-2 nm micropores. Here, we address these issues with two new strategies. First, we show that traditional solution-state NMR can be used to directly monitor polymer chains and quantify their diffusivities as they thread into microporous particles from the surrounding solvent. This label-free, high-resolution, in situ monitoring is possible because once a polymer chain enters the particles, its NMR signal becomes invisible due to excessively slow molecular tumbling. Second, we show that threading rates can be tuned across at least 6 orders of magnitude—without changing the particle size, micropore topology, or polymer chain length—by rationally modifying the particles’ external surface with different coatings that form via noncovalent self-assembly after simple mixing. The strategies described here to quantify and control polymer threading are simple and generalizable to a wide range of polymer/particle combinations, solvents, temperatures, and concentrations; thus, they may spark advances in diverse technological and fundamental areas that rely on blends of polymers with microporous particles.

## Graphical abstract

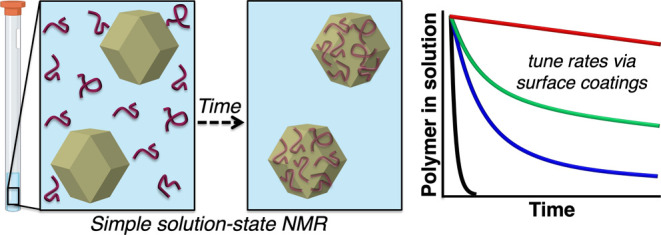

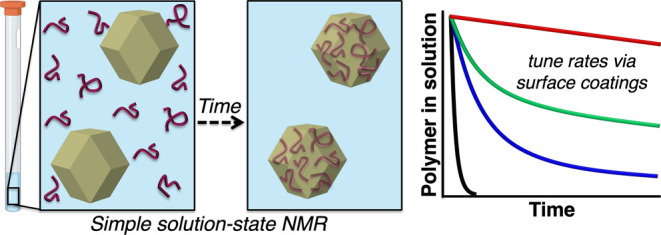

## Introduction

Many chemical,^1–4^ biomedical,^5,6^ and materials^7–9^ technologies now rely on blends of polymers with microporous particles. However, the full potential of these blends is currently limited by the lack of simple and versatile strategies to either quantify or control how polymer chains thread into the sub-2 nm micropores. For one class of technologies—such as mixed-matrix membranes and porous liquids—it is preferable to prevent polymer chains from filling up micropores so that the particles retain their traditional functionality of separating or storing gas molecules.^1,2,5,7^ A common approach to preserve empty micropores is to use rigid, bulky polymers that simply cannot fit inside. Yet relying solely on such polymers greatly narrows the design space and performance of these systems by excluding flexible, sterically lean polymers that would otherwise offer valuable properties (such as toughness or elasticity for membranes and biocompatibility or viscosity for porous liquids). Conversely, for another class of technologies—which include catalysts to recycle polymers,^3^ compartments to separate heteropolymers,^10^ and additives to improve mechanical properties^9^—it is necessary for polymer chains to thread into micropores so their conformations and kinetics can be manipulated in unique ways. However, because this class of technologies is in its infancy and polymer/particle combinations are chosen empirically, their properties cannot be precisely tuned. Developing simple and versatile strategies to quantify, understand, and control polymer threading could therefore unlock key advances for microporous systems across diverse technologies.

A major obstacle toward this aim is that current techniques to analyze the behavior of guest molecules in microporous particles have been developed with either gas molecule guests or narrow conditions in mind. For instance, polymers effectively have zero vapor pressure, so the pressure-based sorption measurements that are commonly used to quantify gas uptake and diffusion cannot be adapted to measure the same properties of polymer guests.^11^ Polymer uptake and diffusion have been directly measured (with varying degrees of ease and accuracy) using techniques such as powder X-ray diffraction (PXRD),^12,13^ solid-state nuclear magnetic resonance (NMR) spectroscopy,^14^ or even simply weighing particles before and after incubating them in a polymer solution.^15^ However, PXRD and solid-state NMR have only been used to measure diffusion *in situ* from a solvent-free polymer melt due to practical limitations of the instruments; solid-state NMR and gravimetric approaches offer poor time resolution that cannot capture many relevant time scales for diffusion; and PXRD and gravimetric approaches can give misleading results if solvent molecules are trapped with polymers inside the pores.

Beyond these analytical hurdles, broadly applicable strategies to control polymer threading remain elusive because existing studies focus only on a narrow set of microporous hosts. Most insights derive from single-pore platforms—such as those used for DNA sequencing^16^—or particles whose pores form isolated one-dimensional channels.^10,13,15^ In contrast, many common metal–organic frameworks,^17^ zeolites,^18^ and covalent–organic frameworks^19^ feature networks of micropores that are interconnected in three dimensions. Inside such networks, polymer chains longer than ∼100 monomers must occupy multiple pores simultaneously since each micropore holds only a few tens of monomers. The conformational and translational restrictions that arise from this confinement introduce kinetic, entropic, and geometric considerations that may give rise to unique properties relative to polymers in isolated pores or pore channels. Existing efforts also rely on native, unmodified particles and thus ignore how modification of the external surface can gate the entry of polymers without changing the underlying micropore framework or environmental conditions. Therefore, expanding the design space to include diverse frameworks and surfaces offers a clear path to rationally control polymer threading.

Here we introduce two complementary strategies to quantify and control polymer threading in particles with interconnected micropores (Figure 1). We first adapt traditional solution-state NMR to track polymer chains in real time as they thread from the bulk solvent into the pore network. This approach allows for label-free determination of the effective diffusivities of polymer threading using a widely accessible tool. We then show that threading rates can be tuned by rationally modifying the particles’ exterior via noncovalently self-assembled coatings. Varying the surface chemistry alone allows the effective diffusivities of threading to span at least 6 orders of magnitude (from ∼10^–15^ to ∼10^–21^ m^2^ s^–1^) without changing the particle size, micropore topology, or polymer chain length. The strategies introduced here to quantify and control polymer threading are simple and generalizable to a wide range of particle/polymer combinations and experimental conditions such as temperature, solvent, and concentration. Thus, these strategies may spark significant advances for understanding and engineering blends of polymers with microporous particles.

**Figure 1:**
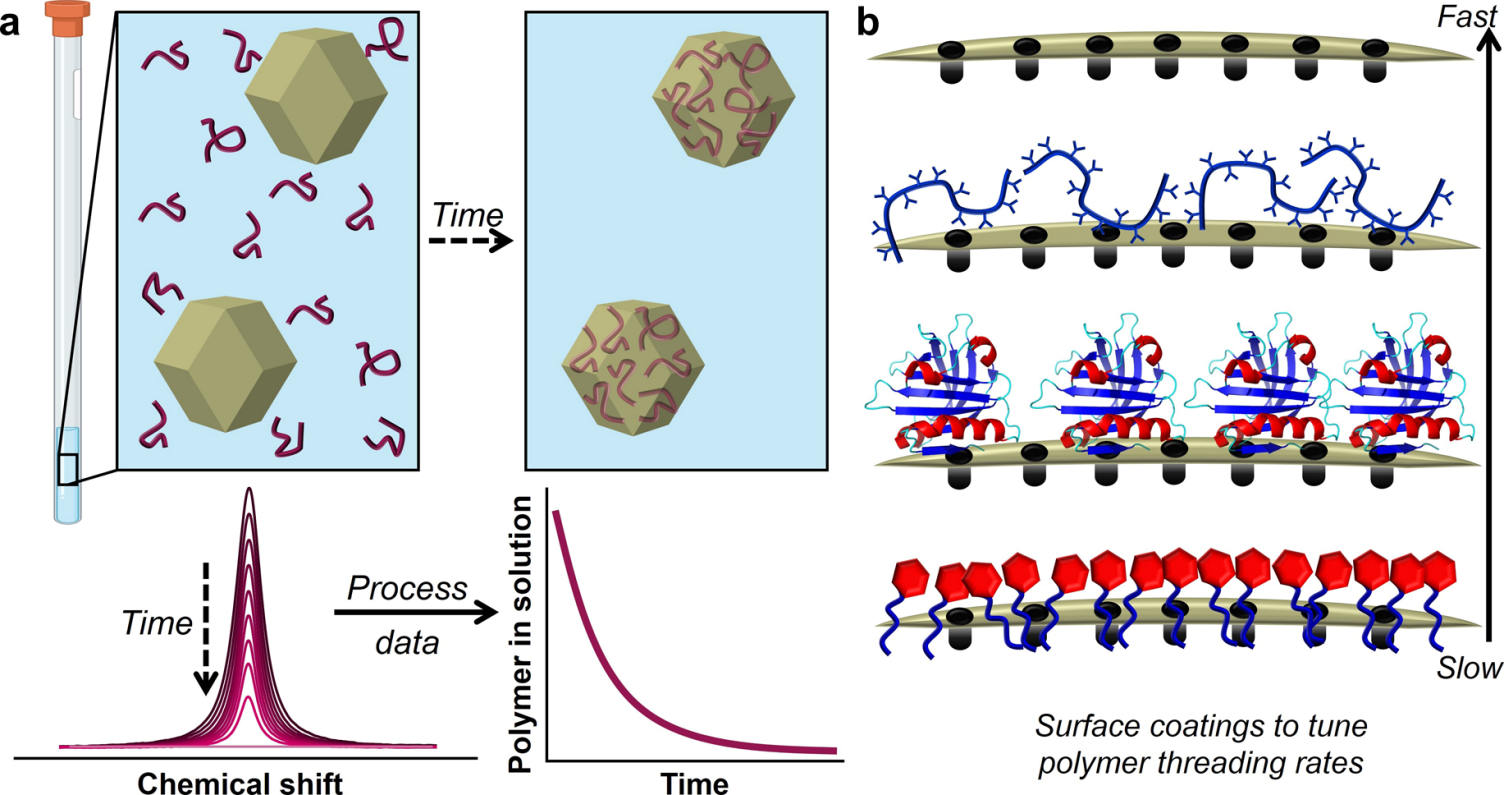
Schematic illustration of (a) the solution-state NMR technique we developed here to monitor and quantify the threading of polymers into microporous particles, and (b) the noncovalent surface functionalization strategy to tune the threading rates of polymers.

## Results and Discussion

### Solution-State NMR as a Simple Tool to Quantify Polymer Threading into Microporous Particles

We hypothesized that solution-state NMR could serve as an *in situ*, label-free method to quantify polymer threading into microporous particles. Specifically, it is well established that relatively larger nanoparticles (over ∼5 to 10 nm) have excessively slow rates of molecular tumbling in solution. This causes extreme line broadening in their NMR signals that essentially makes nanoparticles—and any attached organic ligands—invisible in solution-state NMR (Figure S1).^20^ It follows that polymer chains inside microporous particles may also become invisible because they would tumble at similarly slow rates as the confining particles due to extreme confinement in narrow pores. This phenomenon could then be exploited to measure the rate at which polymer chains thread from the solvent (where they are visible to solution-state NMR) to inside microporous particles (where they are invisible).

To test this hypothesis, we first used a model system of zeolitic imidazolate framework-8 (ZIF-8, Figures S2–S5) as the microporous nanoparticle,^21^ polyethylene glycol (PEG) as the polymer, and deuterated water as the solvent. ZIF-8 has a large pore volume (∼0.6 cm^3^/g) and surface area (∼1,500 to ∼2,000 m^2^/g) (Figure S5),^21,22^ allowing ZIF-8 particles to take up large quantities of guest molecules. Further, ZIF-8 is made up of micropores with 1.1 nm diameters that are interconnected in three dimensions by flexible apertures with up to ∼0.7 nm diameters,^21–24^ so PEG chains are sterically lean enough to thread through the pores after uncoiling if the given conditions make that process thermodynamically favorable.^25^ Blends of PEG/ZIF-8 are relevant in diverse technologies, including drug delivery,^26,27^ porous liquids,^5,28^ and mixed-matrix membranes.^29,30^ Notably, each of these examples requires very different processing and usage conditions, and some have shown PEG/ZIF-8 blends that have empty pores^26,30^ while others have shown polymer-filled pores.^5,28^ These previous examples demonstrate that different conditions—such as solvent, polymer length, and temperature—can dramatically alter the extent and rate at which polymers thread into micropores, further reinforcing the importance of developing strategies to fundamentally understand and control polymer threading.

We first dispersed bare ZIF-8 nanoparticles with an average particle size of 240 nm ± 10 nm in deuterated water at a concentration of 20 mg/mL, achieving good dispersibility through rigorous ultrasonication (Figures S6 and S7). We then added PEG to the dispersion to reach a final concentration of 3 mg/mL PEG and 10 mg/mL ZIF-8. We chose these experimental conditions for two reasons: (i) 3 mg/mL is within the dilute regime of PEG in water for all molecular weights tested here,^31^ so interchain interactions in the bulk solvent are absent, and (ii) ZIF-8 has a maximum uptake capacity of approximately 4 mg of PEG for every 10 mg of ZIF-8 (Table S1), so all PEG chains can eventually thread into ZIF-8.

After mixing PEG with ZIF-8 in an NMR tube, the intensity of the PEG hydrogen signal decreases rapidly and continuously as a function of time (Figure 2a). For PEG with a molecular weight of 35 kDa, the signal almost completely disappeared within 450 s. As proof that the continuous reduction of PEG’s NMR signal is due to chains threading into the pores of ZIF-8, we ran two control experiments. First, PXRD spectra show that the intensity of the 110 diffraction peak of ZIF-8 is significantly reduced relative to the other diffraction peaks after incubating with PEG for 450 s (Figure 2b). This intensity reduction for the lowest order diffraction peak—without any changes in particle crystallinity, shape, or size (Figure S8)—is known to occur in crystalline microporous materials when disordered molecules enter pores that are initially empty and reduce the scattering contrast between the framework and pore interior.^12,13,28,32^ Since it is known that the micropores of ZIF-8 are hydrophobic enough to prevent water molecules from entering,^5^ the guest molecules must be PEG. Second, we measured the *in situ* oxygen carrying capacity of dispersions made with pure ZIF-8 and PEG/ZIF-8 using a recently developed technique based on an electrode sensor.^5,28^ This electrode approach correlates the gas storage capacity of a dispersion to the amount of micropores that remain empty, and thus can be used to prove whether polymer chains fill ZIF-8 micropores in the experimental conditions of interest (i.e., in water). Dispersions made with pure ZIF-8 had 87% ± 5% of the theoretical oxygen carrying capacity that would be achieved for completely empty ZIF-8 particles (Figure 2c). This result expectedly shows that most of the ZIF-8 micropores remain dry and empty in water. Contrarily, PEG/ZIF-8 dispersions had 7% ± 3% of the theoretical oxygen carrying capacity, which shows that PEG chains occupy the internal micropores of ZIF-8 and prevent them from storing oxygen. These experiments collectively prove that the reduction—and eventual disappearance—of PEG’s NMR signal corresponds to polymer chains threading from the bulk solvent to inside the micropore network of ZIF-8.

**Figure 2:**
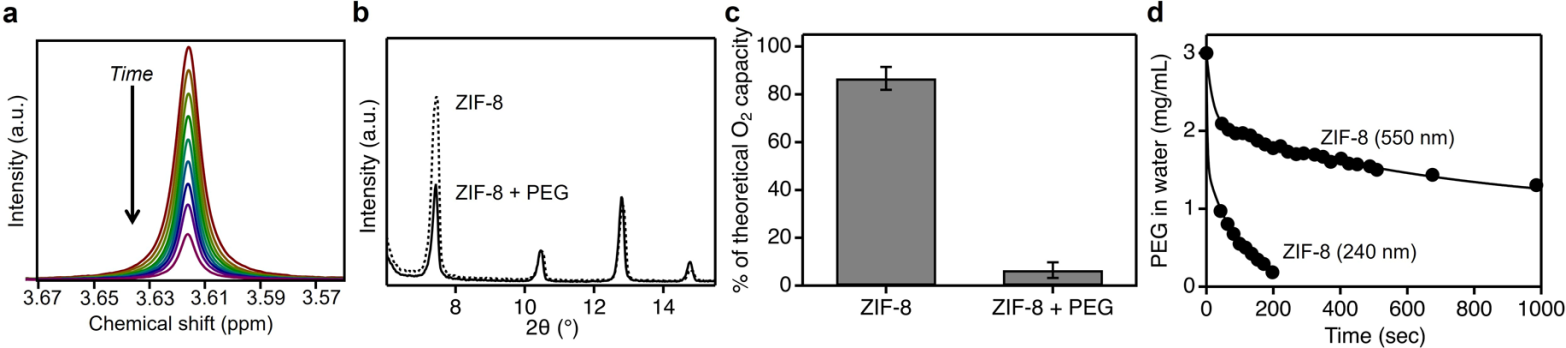
(a) Solution-state NMR spectra of ZIF-8 with PEG (35 kDa) over time; (b) normalized PXRD patterns of pure ZIF-8 and ZIF-8 mixed with PEG; (c) O_2_ carrying capacities for ZIF-8 dispersions with and without PEG in water; (d) concentration of PEG (35 kDa) in bulk solvent over time after mixing with ZIF-8 nanoparticles (both data sets are fit to a double exponential decay).

We then used NMR to quantify the effective diffusion coefficient for PEG threading into bare ZIF-8 and obtain fundamental insights into polymer threading. By using Pulse length based concentration determination NMR spectroscopy (PULCON),^33^ the absolute integral of the PEG signal at a given time can be directly converted to the amount of PEG remaining in the bulk solvent without the need for an internal reference molecule in the NMR tube (whose presence could interfere with polymer threading). For PEG 35 kDa, the diffusion process appears to fit a double exponential decay rather than an exponential or logarithmic decay (Figure 2d, S9, S10). The “fast” and “slow” diffusion steps have effective diffusion coefficients of 1.0 × 10^–15^ ± 3.5 × 10^–16^ m^2^ s^–1^ and 1.6 × 10^–17^ ± 1.6 × 10^–18^ m^2^ s^–1^, respectively. The threading rate is significantly affected by chain length: shorter chains (PEG 2 kDa) thread much faster than PEG 35 kDa, while longer chains (PEG 200 kDa) thread more slowly (Figure S11). Further, when the particle size of ZIF-8 is increased by ∼2.3× (from 240 nm ± 10 to 550 nm ± 55 nm), and the mass concentration of ZIF-8 is held constant at 10 mg/mL, the “fast” and “slow” effective diffusion coefficients for threading of PEG 35 kDa slowed down by a factor of between 2× and 3×. Notably, diffusion of PEG chains from water to the external surface of ZIF-8 particles is not rate-limiting, as the self-diffusivity of PEG in water is at least 4 orders of magnitude faster than the effective diffusivity of PEG threading for a given molecular weight (Figure S12).

Based on these insights, and by considering ZIF-8 as spherical particles made up of concentric layers of micropores, we ascribe the double exponential decay of PEG threading to a two-step process. PEG chains first rapidly fill up a certain amount of pore layers nearest to the external surface inside ZIF-8 particles, and then threading deeper into the particles becomes hindered as the number of accessible micropores shrinks closer to the particles’ core. This model is supported by the dependence of diffusivity on ZIF-8 particle size. Specifically, since the mass concentration of ZIF-8 was held constant, the number of particles (*N*) scales with the particle radius (*r*) as *N* ∼ *r* ^–3^ while the surface area per particle (SA) scales as SA ∼ *r* ^2^. As such, if there is a rate-limiting step that depends on polymer entry into the particle at or near the external surface, effective diffusivities should depend on *r* ^–1^ (i.e., the product of *N* times SA), which is approximately what we observed. Future insights will refine and enhance this proposed model by quantifying the conformations, segmental mobility, and thermodynamic considerations of polymer chains inside micropore frameworks. Overall, the data and analysis shown here clearly demonstrate that solution-state NMR can serve as a simple, widely accessible, high-throughput tool to directly quantify the extent and rate of polymer threading into microporous particles *in situ* with high temporal resolution.

### Noncovalent Modification of the External Particle Surface to Control Polymer Threading

We next sought to develop a generalizable strategy to control polymer threading without changing the particle size, micropore topology, or environmental conditions, since those parameters are often dictated by the design requirements of a specific technology. We hypothesized that the accessibility of entry sites for polymer threading could be modulated in a simple and generalizable way by noncovalently modifying the external surface of the particles.^34,35^ We chose to functionalize ZIF-8 with three different types of molecules: benzalkonium chloride (a small molecule), poly­(*N*-isopropylacrylamide) (PNIPAM, a polymer), and lactoglobulin (a globular protein) (Figure 3a). These molecules are all amphiphilic and should readily adsorb to the hydrophobic ZIF-8 surface by simply mixing them in water. They also should remain pinned to the external surface rather than fill up the internal pore volume due to a combination of steric and energetic considerations.^5,28,36^ Finally, they have different sizes, flexibilities, and chemical compositions, which should lead to differences in packing densities and interactions at the particle surface that may translate to broad control over polymer threading.

**Figure 3:**
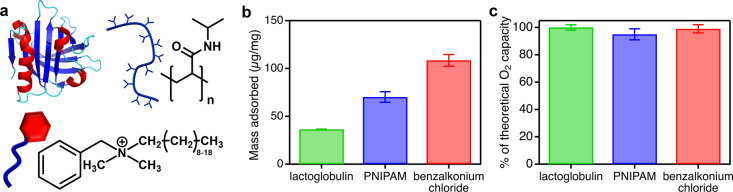
(a) Structures of the molecules used to modify the surface of ZIF-8 nanoparticles: lactoglobulin (top-left), PNIPAM (top-right), and benzalkonium chloride (bottom); (b) mass of molecules adsorbed to the surface of ZIF-8; (c) O_2_ carrying capacities for dispersions of ZIF-8 coated with different molecules.

We confirmed that all three molecules coated the ZIF-8 particles in water (Figure 3b, S13–S15) and remained localized to the external surface rather than filling up the internal pore volume based on both PXRD (Figure S16) and *in situ* oxygen sensor (Figure 3c) experiments. The functionalized ZIF-8 particles remained colloidally stable for at least 24 h (Figure S7) and showed no changes in crystallinity or particle shape and size (Figures S16 and S17). Lactoglobulin showed the lowest adsorbed mass of 36 μg per mg of ZIF-8, presumably because the high charge density and low conformational flexibility of globular proteins prevent them from packing as densely on surfaces as small molecules or synthetic polymers.^37^ If we assume that lactoglobulin adsorbs without global unfolding and packs efficiently—reasonable assumptions for the adsorption of highly structured proteins^37^—we can estimate the average surface coverage based on the known footprint of lactoglobulin (Figure S18). These assumptions lead to an adsorption density of 0.045 protein molecules per nm^2^ and an approximate surface coverage of 51%. Thus, in our experimental conditions, lactoglobulin adsorbs as a loosely packed monolayer on the ZIF-8 surface. The footprint of PNIPAM and benzalkonium chloride cannot be estimated—even though the adsorbed mass can be accurately quantified—since flexible polymers and small molecules can rearrange dramatically when they adsorb.^37–39^ However, based on their higher adsorbed mass density and greater conformational flexibility, we assume that PNIPAM and benzalkonium chloride cover more of the ZIF-8 surface than lactoglobulin.

Using our NMR approach, we quantified the diffusion mechanism and rates for PEG threading into surface-coated ZIF-8. For both “short” (2 kDa) and “long” (35 kDa) chains and all three surface coatings, PEG threading follows a first-order exponential decay and is rate-limited by diffusion at the external surface (Figure 4 and S19–S23).^24^ This surface-limited diffusion model confirms our hypothesis that access to the particles’ entry sites at the external surface is an important factor in polymer threading. Excitingly, the rate of PEG threading differs dramatically depending on the external surface coating of ZIF-8 (Table 1). PNIPAM and lactoglobulin coatings slow down PEG threading by 4 orders of magnitude relative to bare ZIF-8, while benzalkonium chloride coating slows down PEG threading even more (over 6 orders of magnitude relative to bare ZIF-8).

**Figure 4:**
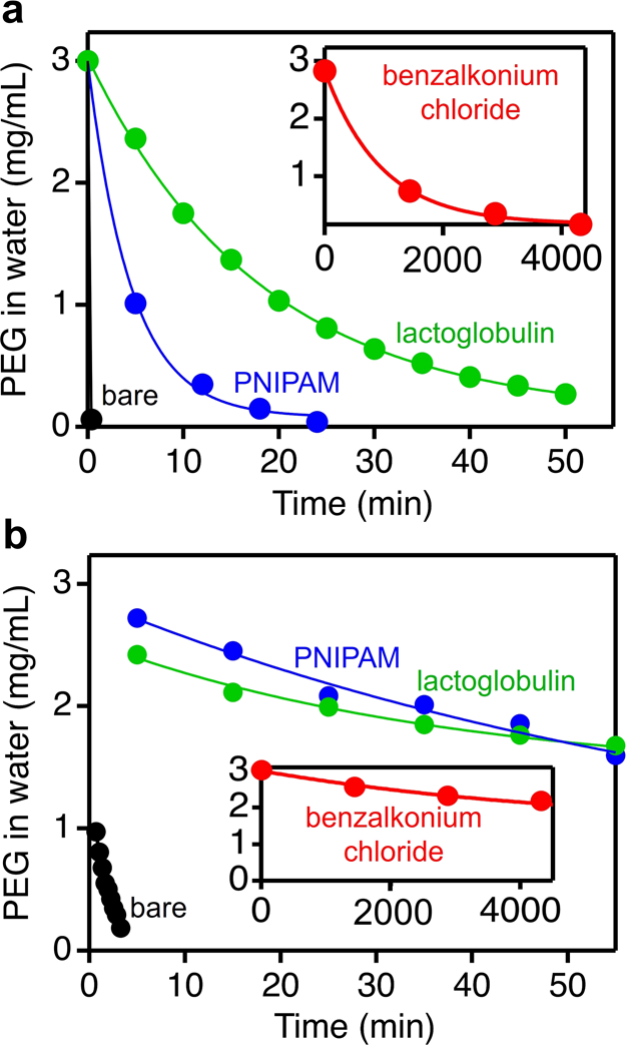
Representative curves for (a) PEG 2 kDa and (b) PEG 35 kDa threading into surface-functionalized ZIF-8 particles.

**Table 1:** Effective Diffusion Coefficients (*D*_eff_) for PEG Threading into ZIF-8 with Different Surface Coatings

ZIF-8 surface coating	*D*_eff_ of PEG 2 kDa (m^2^ s^–1^)	*D*_eff_ of PEG 35 kDa (m^2^ s^–1^)
**No coating** (bare ZIF-8)	>10^–15^	1.0 × 10^–15^ ± 3.5 ×10^–16^ (fast); 1.6 × 10^–17^ ± 1.6 × 10^–18^ (slow)
**PNIPAM**	3.5 × 10^–18^ ± 5.2 ×10^–19^	2.0 × 10^–19^ ± 9.6 × 10^–20^
**Lactoglobulin**	1.1 × 10^–18^ ± 1.3 × 10^–19^	1.5 × 10^–19^ ± 8.4 × 10^–20^
**Benzalkonium chloride**	1.9 × 10^–20^	3.4 × 10^–21^

Polymer threading rates do not show a clear dependence on the total mass of surface-adsorbed coatings, which indicates that differences in chemical structure and packing density of the coatings likely play a dominant role. As the most striking example, the adsorbed mass density of benzalkonium chloride is only ∼1.5× greater than that of PNIPAM, yet PEG threads ∼60× slower into ZIF-8 with benzalkonium chloride coatings than with PNIPAM coatings. We propose two potential explanations. First, the “effective miscibility” of PEG with the different coatings may account for differences in threading rates, since PEG chains must pass through the coating to access the pore framework. Indeed, PEG is likely more miscible with PNIPAM than benzalkonium chloride, given their respective chemical compositions. Second, the aliphatic tail of benzalkonium chloride can transiently plug pores that serve as entry sites at the external surface because the tail is flexible and sterically lean. This transient plugging of entry sites could significantly slow down the entry rates of polymer chains into ZIF-8 compared with coatings of lactoglobulin and PNIPAM, which are much bulkier and thus cannot easily plug the entry sites (if at all). These insights clearly show that noncovalent surface modification using molecules with diverse physicochemical features is an effective and simple strategy to mediate polymer threading into microporous particles.

### Generalizability of New Approaches to Quantify and Control Polymer Threading

Finally, we implemented our new approaches across a wide range of experimental conditions to prove that they are generalizable and to obtain deeper insights into polymer threading. First, we blended PEG 2 kDa (3 mg/mL) with ZIF-8 (10 mg/mL) in different deuterated solvents for 24 h and then ran NMR on the blends *in situ*. The NMR signal of PEG only disappeared when water was the solvent, while no change was observed in methanol, dimethyl sulfoxide (DMSO), and chloroform (Figure 5a and S24). These data show that PEG threads into ZIF-8 only in water but remains freely dissolved outside the particles in the other three solvents. As a control to ensure the validity of this interpretation, we centrifuged PEG/ZIF-8 blends dispersed in methanol or water to separate ZIF-8 particles from any polymers that remained freely dissolved and then checked the PEG concentration in the supernatant. No change occurred from the initial PEG concentration for the supernatant in methanol, while all the PEG was removed from the supernatant in water (Figure S25). Further, when PEG-filled ZIF-8 particles were removed from water, dried, and redispersed in methanol, the PEG chains rapidly threaded out of the particles into the bulk solvent (Figure S26). These experiments show that solvents play a key role in determining the thermodynamic driving force of polymer threading for a given polymer/particle combination. They also show that NMR can serve as a useful tool to screen whether a polymer threads into a microporous particle in different solvents.

**Figure 5:**
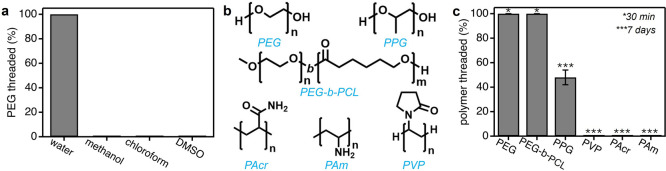
(a) Amount of PEG threaded from bulk solvent into ZIF-8 particles in different deuterated solvents; (b) chemical structures of different polymers used in this study; (c) amount of polymer threaded from bulk water into ZIF-8 at different specified times.

Next, we measured polymer threading into ZIF-8 for polymers with diverse backbones, side groups, and architectures (Figure 5b,c), with water as the solvent. Several interesting phenomena were observed. For instance, a diblock copolymer made of PEG and poly­(caprolactone) (PEG-*b-*PCL) threads at similarly rapid rates as pure PEG of the same length, and all the polymer chains have diffused from water to inside ZIF-8 within 5 min (Figures S27 and S28). PCL is a flexible polyester with a narrow cross-sectional diameter, so this result is perhaps not surprising. However, polypropylene glycol (PPG), a homopolymer of similar length, threads at substantially slower rates—only ∼half of the polymer chains have threaded from water to inside ZIF-8 after 7 days (Figures S28 and S29). PPG and PEG are identical polymers except that PPG has one methyl side group in each repeat unit. It is feasible that this seemingly minor increase in steric bulk substantially raises the activation barrier for a segment to pass through the narrow ZIF-8 apertures, as has been observed for the diffusion of bulky gas molecules that differ by only a few atoms.^23^ Finally, for three homopolymers with polyolefin backbones but different polar side groups, none of the polymer chains showed any signs of threading into ZIF-8 after 7 days, either with NMR or control PXRD experiments (Figures S30–S33). We attribute this to steric hindrance (for polyvinylpyrrolidone (PVP) and polyacrylamide (PAcr)) and unfavorable energetics (for polyvinyl amine (PAm)). We emphasize that the polymers included here are not intended to be exhaustive but rather serve as representative examples to demonstrate that our approach is applicable to a wide range of diverse polymers.

Polymer threading can also be quantified and controlled over a wide range of concentrations and temperatures. For instance, polymer concentrations can be quantified over a range of at least 0.05 to 30 mg/mL, as shown using PEG/ZIF-8 in water as a model system (Figure S34). The true upper limit on polymer concentration is likely higher and only occurs when solutions become too viscous for the polymer chains to relax quickly enough for accurate detection in solution-state NMR. This limit will vary across different systems because it depends on the critical entanglement concentration, which itself depends on polymer length, interchain interactions, and solvent quality. Additionally, temperature can be used as another handle to control threading rates. The rate at which PEG threads into ZIF-8 coated with benzalkonium chloride was substantially increased when the temperature was raised from 20 to 40 °C (Figure S35). These experiments clearly demonstrate that our strategies to quantify and control polymer threading are broadly applicable across diverse experimental conditions, which should facilitate their translation into both fundamental and applied research.

### Outlook

We anticipate that the strategies and insights described here can be used to spark important advances in diverse areas ranging from fundamental polymer science to chemical, biomedical, and materials technologies. For instance, as demonstrated in this article, solution-state NMR can be used as a widely accessible, label-free, high-throughput tool to screen and quantify the extent and rate of polymer threading into sub-2 nm micropores for different polymer/particle combinations in different solvents, temperatures, and concentrations. This information can speed up the optimization of both liquid and solid-state technologies by facilitating the selection of polymer/particle combinations and refining the processing or usage conditions. The fundamental insights obtained from such experiments may also lead to broader design rules that clarify which features govern polymer threading in different micropore frameworks. Further, the ability to control polymer threading rates over at least 6 orders of magnitude using a quick and simple noncovalent functionalization strategy should also be broadly impactful, since the only design requirement is that the functionalizing molecules cannot fill up the particles. This requirement leaves many small molecules, polymers, and—for aqueous applications—proteins to choose from. Such control may eventually allow sterically lean polymers to be used as supporting matrices or additives in technologies where pore filling is undesirable (if the coating sufficiently slows or arrests polymer threading altogether, for instance). It may also introduce opportunities to access a wider range of properties in technologies where pore filling is required. Finally, the quantification and control strategies described here offer exciting opportunities to explore and exploit fundamental polymer physics at the single-chain level.

## Supplementary Material



## Notes

Detailed experimental procedures, characterization, and additional supporting data (Table S1 and Figures S1–S35) (PDF)

## Abbreviations

PEG: polyethylene glycol
ZIF-8: zeolitic imidazolate framework-8
PNIPAM: poly­(*N*-isopropyl acrylamide)
PEG-*b*-PCL: poly­(ethylene glycol-*b*-caprolactone)
PPG: polypropylene glycol
PVP: polyvinylpyrrolidone
PAcr: polyacrylamide
PAm: polyvinyl amine
PXRD: powder X-ray diffraction
NMR: nuclear magnetic resonance
PULCON: pulse length based concentration determination NMR spectroscopy
