# Supplementary material for: Simple Strategies to Quantify and Control Polymer Threading into Micropores
Source: J Am Chem Soc. 2025 Jun 9;147(25):22245–52. doi: 10.1021/jacs.5c07556 (PMC12203582; doi:10.1021/jacs.5c07556)
Supplement: Supplementary file 1 [file ja5c07556_si_001.pdf]

Supporting Information for:

# Simple Strategies to Quantify and Control Polymer Threading into Micropores

Supreet Kaur,[a] Benjamin Lesea-Pringle,[a,b] Surya Marjit,[a] Hyukhun Hong,[c] Aqib Rahman,[a] Boran Ma,[d] Denize C. Favaro,[e] and Christopher DelRe[a,b,f]*

[a]Nanoscience Initiative, CUNY Advanced Science Research Center, New York, New York, 10031 USA

[b]Ph.D. Program in Biochemistry, The Graduate Center of the City University of New York, 365 5th Ave, New York, New York, 10016 USA

[c]Department of Chemistry and Chemical Biology, Harvard University, Cambridge, Massachusetts, 02138 USA

[d]School of Polymer Science and Engineering, University of Southern Mississippi, 118 College Drive, Hattiesburg, Mississippi, 39406 USA

[e]Structural Biology Initiative, CUNY Advanced Science Research Center, New York, New York, 10031 USA

[f]Department of Chemistry and Biochemistry, City College of New York, 275 Convent Ave, New York, New York, 10031 USA

*J. Am. Chem. Soc.*

## 1. Materials:

*Polymers*: Polyethylene glycol (PEG) of all molecular weights—2, 10, 35, and 200 kDa —and polypropylene glycol (PPG) 2 kDa were purchased from Sigma-Aldrich and used as received. Polyvinylpyrrolidone (PVP) 10 kDa, polyacrylamide 10 kDa, polyvinylamine 9 kDa, and poly(ethylene glycol–b–caprolactone) (PEG–b–PCL) 2–b–2.6 kDa were purchased from Polymer Source, Inc. and used without further purification.

*ZIF-8 synthesis*: We synthesized ZIF-8 nanoparticles with two different average particle sizes. For the smaller particles, we followed a procedure adapted from a previous report.[1] $Zn(NO_3)_2{\cdot}6H_2O$, 2-methylimidazole, and methanol were purchased from Sigma-Aldrich and used as received. The synthesis procedure from that report, followed exactly, is quoted below:

"$Zn(NO_3)_2{\cdot}6H_2O$ (2.94 g, 9.88 mmol) and 2-methylimidazole (6.49 g, 79.0 mmol) were each dissolved in 50 mL of methanol. The two solutions were then mixed rapidly and stirred at 500 RPM for 1.5 h at room temperature. To remove excess reagents after completion of each reaction, the solution was subjected to 1 centrifuge cycle (25 min, 7197 rcf) to remove the reaction solvent and 3 subsequent centrifuge cycles (25 min, 7197 rcf) to perform methanol exchanges. After each cycle, the supernatant was decanted, and the remaining nanocrystals were redispersed in fresh methanol. The particle size of each ZIF-8 sample was determined by measuring the diameter of at least 50 different ZIF-8 particles by scanning electron microscopy." This synthesis procedure led to nanoparticles whose average size varied from batch to batch but stayed in a narrow range from ~240 nm to ~280 nm.

For the larger particles, we followed the procedure adapted from the same previous report:[1]

"$Zn(NO_3)_2{\cdot}6H2O$ (1.76 g, 5.92 mmol) was dissolved in 55 mL of methanol, and 2-methylimidazole (1.95 g, 23.8 mmol) and sodium formate (0.41 g, 6.03 mmol) were dissolved in 55 mL methanol separately. The latter solution was poured into the former solution, and the mixed solution was left without stirring for 14 h at room temperature. To remove excess reagents after completion of each reaction, the solution was subjected to 1 centrifuge cycle (25 min, 7197 rcf) to remove the reaction solvent and 3 subsequent centrifuge cycles (25 min, 7197 rcf) to perform methanol exchanges. After each cycle, the supernatant was decanted, and the remaining nanocrystals were redispersed in fresh methanol. The particle size of each ZIF-8 sample was determined by measuring the diameter of at least 50 different ZIF-8 particles by scanning electron microscopy." This synthesis procedure led to nanoparticles whose average size varied from batch to batch but stayed in a narrow range from ~515 nm to ~550 nm.

*Surface coating molecules*: β-lactoglobulin from bovine milk, amine-terminated poly(N-isopropylacrylamide) (PNIPAM) 5.5 kDa, and benzalkonium chloride were all purchased from Sigma-Aldrich and used as received.

*NMR reagents*: Sodium 3-(trimethylsilyl)-1-propanesulfonate (DSS) and deuterium oxide ($D_2O$) were purchased from Cambridge Isotope Laboratories, Inc. Dimethylsulfoxide-d6 (DMSO-d6), methanol-d4 (MeOD), and chloroform-d ($CDCl_3$) were purchased from Sigma-Aldrich. DSS was used as a reference molecule in certain experiments, as described in the methods section below.

## 2. Characterization of ZIF-8 nanoparticles:

*Scanning electron microscopy:* We performed scanning electron microscopy (SEM) on a FEI Helios NanoLab 660 to obtain high-resolution images of all the synthesized batches of ZIF-8 nanoparticles. Before the sample preparation, ZIF-8 was dried under a vacuum for 2 h. We deposited the crystals on a conductive carbon adhesive tape mounted on an aluminum pin stub. To facilitate imaging and reduce charging, we sputter-coated the samples with a 5-8 nm layer of gold (Au) with the Leica EM ACE600 Coater. The images were analyzed using ImageJ software.

*Transmission electron microscopy:* Carbon-coated grids were purchased from Electron Microscopy Sciences. We drop-casted the sample solution (2 µl) on the carbon-coated grid, removed the excess solution by blotting the grid with filter paper, and left the grid to air dry. The sample was imaged in an FEI Spirit 120kV TEM. The images were analyzed using ImageJ software.

*Powder X-Ray Diffraction:* All powder X-Ray diffraction (PXRD) measurements were obtained on a PANalytical X'Pert Pro using CuKα radiation. We prepared the PXRD samples by freeze-drying the ZIF-8 dispersions and the Polymer/ZIF-8 blends for at least 24 hours, then placed the dried powder on a sample holder to record the PXRD patterns. For experiments where the relative intensities of low-angle peaks were compared to determine whether the ZIF-8 pores remained empty or had guest molecules inside, the data were normalized to allow for direct comparison.

*Brunauer-Emmett-Teller (BET) surface area analysis:* We measured the porosity of ZIF-8 using two distinct batches (both with an average particle size of 240 nm) using a Micromeritics ASAP 2020 PLUS Adsorption Analyzer. We collected adsorption-desorption data using nitrogen ($N_2$) at 77 K. We analyzed the data using BET theory over two pressure ranges: a wide range of $P/P_0$ = 0.009 to 0.1 (which is how ZIF-8 was characterized after its initial discovery[2]) and a narrow range of $P/P_0$ = 0.0049 to 0.035 (which is the range over which both BET criteria are satisfied; specifically, the C constant is positive and $Q \cdot (1-P/P_0)$ monotonically increases over the entire pressure range, where Q is the quantity of $N_2$ in volume.[3,4] As shown in **Figure S5**, the fits are highly linear across all pressures, with $R^2$ values exceeding 0.99. For both pressure ranges, the pore volume was 0.61 $cm^3/g \pm 0.02\ cm^3/g$ and the pore width distribution centered on values below 1.5 nm. For data fit over the wider pressure range, the surface area was 1,860 $m^2/g \pm 70\ m^2/g$; for data fit over the narrower pressure range, the surface area was 2100 ± 60 $m^2/g$. These pore volume, pore width, and surface area values are highly consistent with previously-reported values for ZIF-8.[2, 3, 5] We note that over the wider pressure range ($P/P_0$ = 0.009 to 0.1), the C constant is positive (which satisfies BET criterion 1) but the value $Q \cdot (1-P/P_0)$ does not increase over the entire pressure range (does not satisfy BET criterion 2). However, BET-determined surface area and pore volume values are often reported for microporous materials even when they do not satisfy the BET criterion, as it is well established that gas uptake behavior deviates from ideal cases when the pore size becomes small (as it does for micropores).[3, 6] Indeed, this deviation from BET criteria has been observed before for ZIF-8.[2]

*Solution-state NMR:* All 1-D $^1H$ NMR experiments were conducted on a Bruker Avance III HD spectrometer at 300 MHz ($^1H$ Larmor frequency) equipped with a Bruker Multinuclear Broadband Fluorine Observe (BBFO) probe. We performed all the experiments at 25 ˚C in 5 mm tubes and using Topspin 3.7.0 for spectrometer control.

*In-situ oxygen storage capacity measurements:* We followed an existing procedure to quantify the oxygen ($O_2$) storage capacity of ZIF-8 dispersions in water.[1, 3] To evaluate the gas carrying capacity of ZIF-8 dispersions in water, we measured $O_2$ release from bare and surface-functionalized ZIF-8 dispersions into deoxygenated water using a Unisense MicroRespiration $O_2$ microsensor. Nanopure water was first deoxygenated by sparging with $N_2$ for at least 30 min. Prior to each measurement, the $O_2$ sensor was calibrated using a two-point method: in fully nitrogentated water (0 mg/L $O_2$) and air-equilibrated nanopure water (the known $O_2$ solubility) at a known temperature. Note that temperature was continuously recorded by measuring the temperature of water in an adjacent vial. For measurements, an aliquot of deoxygenated water was sealed in an airtight chamber with a glass stir bar, and a baseline $O_2$ concentration was recorded. A known volume of an air-equilibrated ZIF-8 dispersion was then injected, and the subsequent change in $O_2$ concentration was monitored to calculate the amount of $O_2$ released into water. The amount of $O_2$ released was compared to the theoretically expected amount based on the injected dispersion volume, concentration, and the solid-state $O_2$ adsorption capacity of ZIF-8.[1, 3]

## 3. Dispersing bare ZIF-8 and surface functionalized ZIF-8 particles in water:

We used ultrapure water from a Milli-Q IQ 7000 water purification system for all experiments. We checked all the dispersions under an optical microscope to confirm they were well dispersed; we also compared the dispersions we used for all threading experiments against an intentionally aggregated control dispersion, as shown in **Figures S6**, **S7**.

*Bare ZIF-8 dispersions*: We dried ZIF-8 (20 mg) with a rotary evaporator and first redispersed the dried powder in a methanol:water mixture (800 µL:160 µL). We subjected that to 1 centrifuge cycle (10 min, 16500 rcf), and decanted the supernatant. Then we redispersed this wet pellet in pure water to reach the desired concentration (10 mg/mL).

*ZIF-lactoglobulin dispersions*: We dried ZIF-8 (10 mg) with a rotary evaporator and redispersed it in a 5 mg/mL lactoglobulin solution in water. We sonicated and vortexed the dispersion for 5 to 10 minutes to achieve good dispersibility in water.

*ZIF-PNIPAM dispersions*: We mixed ZIF-8 and PNIPAM in methanol with the same mass ratio (1:1). We dried the mixture using a rotary evaporator and then redispersed it in water to get a final concentration of 10 mg/mL each. We sonicated and vortexed the dispersion for 5-10 minutes to achieve good dispersibility in water.

*ZIF-benzalkonium chloride dispersions*: We mixed ZIF-8 and benzalkonium chloride in methanol with the same mass ratio (1:1). We dried the mixture using a rotary evaporator and then redispersed it in water to get 10 mg/mL concentration each. We sonicated and vortexed the dispersion for 5-10 minutes to achieve good dispersibility in water.

## 4. Quantification of surface coatings:

To quantify the number of molecules adsorbed to ZIF-8 particles, we prepared the dispersions of

ZIF-8 (10 mg/mL) with lactoglobulin (5 mg/mL), PNIPAM (10 mg/mL), and benzalkonium chloride (10 mg/mL). The concentration of the molecules in the dispersion that are not bound to the surface of the ZIF-8 particles was measured using the following spectroscopic methods:

- We recorded the UV-Vis spectra for pure lactoglobulin (5 mg/mL) in water and the supernatant of ZIF-8 dispersed with lactoglobulin in the ratio of 10:5 mg/mL. We used the adsorption peak at 280 nm to quantify the concentration in both pure lactoglobulin solutions and in the supernatant after centrifuging the ZIF-8 dispersion. The difference in the concentrations of lactoglobulin in water versus in the supernatant of ZIF-8 dispersions was taken to be the amount of lactoglobulin adsorbed to ZIF-8.

- We recorded the solution-state $^1$H NMR spectra in deuterated water for pure PNIPAM (10 mg/mL) and ZIF-8 dispersed with PNIPAM in the ratio 10:10 mg/mL. DSS (1 mg/mL) was used as an internal standard to quantify the integrals of the PNIPAM hydrogens at 3.82 ppm. The difference in the concentrations of PNIPAM in water and the supernatant of the dispersion was taken to be the amount of PNIPAM adsorbed on the ZIF-8 surface. We performed the same $^1$H NMR procedure for ZIF-8 with benzalkonium chloride in a 10:10 mg/mL ratio. The concentration of benzalkonium chloride was calculated corresponding to a 4.38 ppm peak.

## 5. Measurements of PEG threading:

*Infiltration rates for bare ZIF-8:* For the rates measured without surface coatings, we used the zg (90deg) pulse sequence. We used the PULCON technique to perform quantitative (qNMR) measurements and calculate the concentration without an internal standard.[7,8] Fundamental to this technique is the exact tuning and matching of the NMR system before and during each session. We used the Topspin command "atma exact" at the beginning of every session to precisely tune and match the spectrometer, and as needed, the "wobb" command suggested the tuning and matching precision had decreased. Due to the speed of PEG infiltration into ZIF-8 in the absence of surface coatings, we modified the zg pulse program's default parameters in order to get time points as close to the start of the reaction as possible. We verified near-complete relaxation of the PEG spins — the quantitative accuracy, after pulse program modification by the effect on pure PEG samples of known concentrations. We set the acquisition time to 4 seconds, and the recycle delay to 0.01 seconds, for a total 4-second time between 90deg pulses. No steady-state scans were necessary, so we set DS to zero. The high signal-to-noise of the PEG system allowed for one scan per experiment (NS=1), and the receiver gain was set to 32 for all experiments. Pulse length calibration via the pulsecal command across different experiments showed only a few percent difference in the P1 needed for a 90deg pulse, and the pulsecal command takes ~40 seconds to run, so we left pulse lengths for all measurements at the default 15 µs.

The PULCON technique allows the use of almost any chemical species as the external standard. For example, on our spectrometer and probe, during the months-long period in which measurements were performed, different samples of pure PEGs of different molecular weights (ranging from 2 kDa to 200 kDa) consistently showed an absolute integral value of ~70,000 for 3 mg/mL PEG (for a receiver gain of 32 dB and NS=16), though it should be kept in mind that the specific value is expected to be unique to a given NMR and probe system. Both PULCON and DSS qNMR measurements consistently gave PEG concentrations within <10% of the expected

concentration of a given sample.

*Infiltration rates for surface-coated ZIF-8:* For the PEG infiltration rates measured in the presence of surface-coated ZIF-8 particles, we used the default zg30 pulse sequence. We first measured the longitudinal relaxation ($T_1$) of PEG alone in water using the inversion-recovery pulse sequence; we obtained a value of 600 ms. We used the default acquisition time of 5.45 seconds and the default recycle delay (D1) of 1 second, for a total inter-pulse time of 6.45 seconds. To give additional confidence to our quantification, we used sodium 3-(trimethylsilyl)-1-propanesulfonate (DSS) as the quantitative NMR reference at a concentration of 1 mg/mL for calculating PEG concentrations in some experiments with surface-coated ZIF-8.

*Estimating maximum uptake capacity of PEG in ZIF-8:* We measured the maximum uptake of PEG by ZIF-8 particles by adding different concentrations of excess PEG and monitoring the reaction *via* NMR until it plateaued. We found the particles took up approximately 40% on average of their weight in PEG across these three trials (Table S1). Specifically, 10 mg of ZIF-8 in solution can store up to approximately 4 mg of PEG inside its micropore network.

*NMR Data Processing:* We used MestReNova (Version 12.02) from Mestre Lab Research for NMR spectra processing. We applied automatic phasing (followed by manual phasing when needed) to all spectra, followed by automatic baseline correction. We normalized the PEG integrals to DSS to obtain PEG concentrations for the surface-coating reactions. The absolute integral value was used to obtain the PEG concentration for the bare ZIF reactions.

**6. Mathematical quantification of PEG threading:**

*Threading into bare ZIF-8:* We found that a double exponential decay model was clearly the best fit to quantify PEG threading into bare ZIF-8. We extracted the rate constants $k_{fast}$ and $k_{slow}$ from the double exponential fitting in Prism 10 software and calculated the diffusion coefficients $D_{fast}$ and $D_{slow}$ using equation S1:

$$D = \frac{kr^2}{\pi^2} \quad \text{(S1)}$$

where r is the radius of ZIF-8 particles and k is the measured rate constant.

*Threading into surface-functionalized ZIF-8:* To quantify the diffusion coefficients of PEG threading into ZIF-8 particles with different surface coatings, we fit the data with a well-established surface barrier model (equation S2),[9] a type of first-order exponential decay:

$$c_t = c_\infty \left[1 - e^{\left(-\frac{3\alpha}{r}\right)t}\right] \quad \text{(S2)}$$

where $c_t$ is the concentration of infiltrated PEG, $c_\infty$ is the saturated concentration (which for all studies here is 3 mg/mL), $\alpha$ is the surface permeability coefficient, and $r$ is the average particle radius. Note that we controlled for batch-to-batch variability of ZIF-8 particle size when calculating diffusion coefficients (D), which is then given by $D = \alpha r/5$.

7. **De-threading of PEG from the PEG/ZIF-8 blend:**

We checked the reversibility of PEG threading into the ZIF-8 pores by performing a solution-state NMR experiment. We mixed ZIF-8 (10 mg/mL) with PEG 2 kDa (3 mg/mL) in water for 0.5 h, which is sufficient time for all PEG chains to thread inside ZIF-8 at this polymer molecular weight. We lyophilized this PEG/ZIF-8 blend and then redispersed it in deuterated MeOH (MeOD-d4) to record the $^{1}$H-NMR spectra over time. We quantified the concentration of PEG de-threading from ZIF-8 and coming into the bulk solvent over time with the help of PEG absolute integrals. The amount of PEG de-threading into the bulk solvent gradually increased and reached 3 mg/mL after ~30 min, which is the amount of PEG initially taken to make the PEG/ZIF-8 blend.

**Supporting Tables.**

**Table S1.** Uptake of PEG 35 kDa into ZIF-8 particles, measured after incubating for 24 h.

| ZIF-8 concentration (mg/mL) | PEG initial concentration (mg/mL) | PEG final concentration (mg/mL) | PEG uptake (mg/mL) | Uptake capacity of PEG in ZIF-8 (%) |
|---|---|---|---|---|
| 10 | 6 | 2.1 | 3.9 | 39 |
| 10 | 9 | 4.8 | 4.2 | 42 |

**Supporting Figures.**

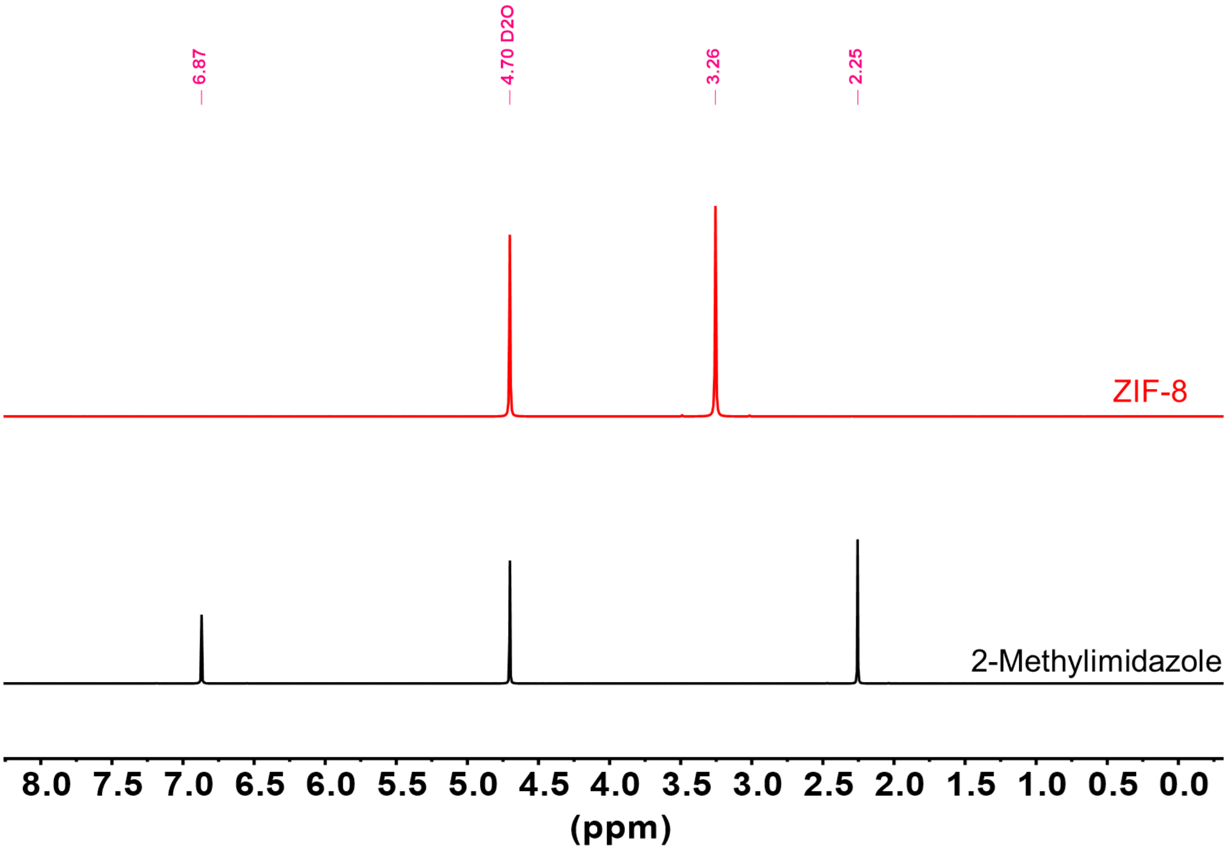


**Figure S1.** Solution-state $^1$H-NMR spectra of ZIF-8 nanoparticles (top) and soluble 2-methylimidazole (bottom), where the effective concentration of 2-methylimidazole is the same in both samples; the peaks of 2-methylimidazole are clearly visible when the molecule is dissolved, whereas they completely disappear when the molecule resides in the structural framework of ZIF-8 nanoparticles due to slow molecular tumbling (note that the 3.26 ppm corresponds to methanol, which was present in this bare ZIF-8 dispersion in water at low concentrations).

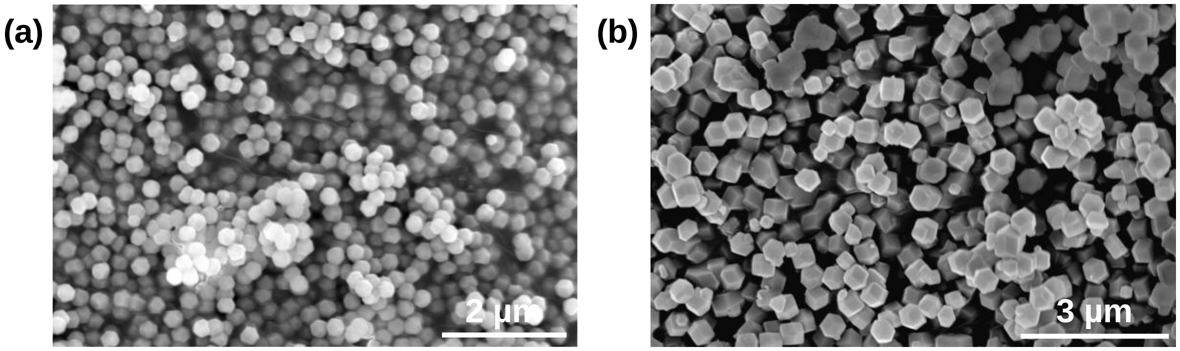


**Figure S2.** Representative SEM images of ZIF-8 particles used throughout the article: (a) ZIF-8 240 nm and (b) ZIF-8 550 nm. We note the presence of a small amount of much smaller particles (<200nm) in the ZIF-8 550 nm batch; however, these smaller particles comprise less than 5% of the total sample by mass, so they were ignored in the particle size calculation.

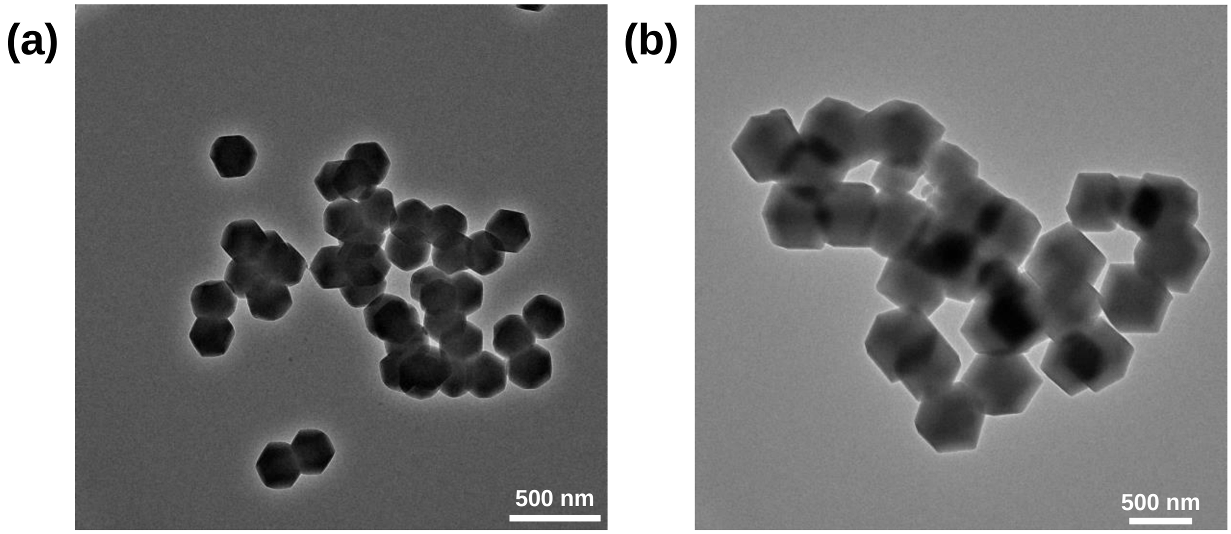


**Figure S3.** Representative TEM images of ZIF-8 particles used throughout the article: (a) ZIF-8 240 nm and (b) ZIF-8 550 nm.

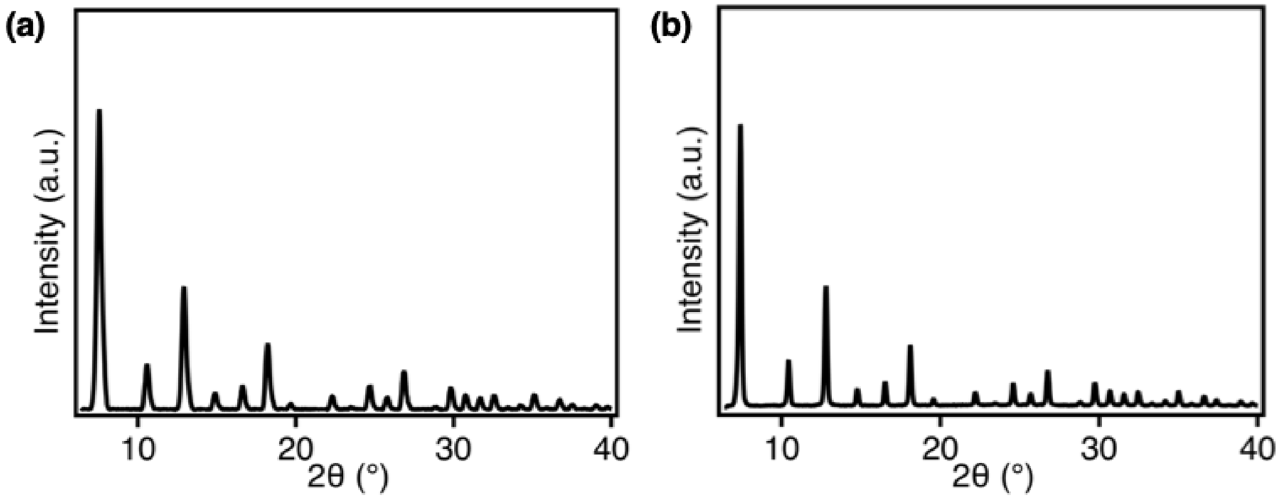


**Figure S4.** Representative powder X-ray diffraction (PXRD) patterns of the synthesized ZIF-8 nanoparticles with different particle sizes: (a) 240 nm and (b) 550 nm.

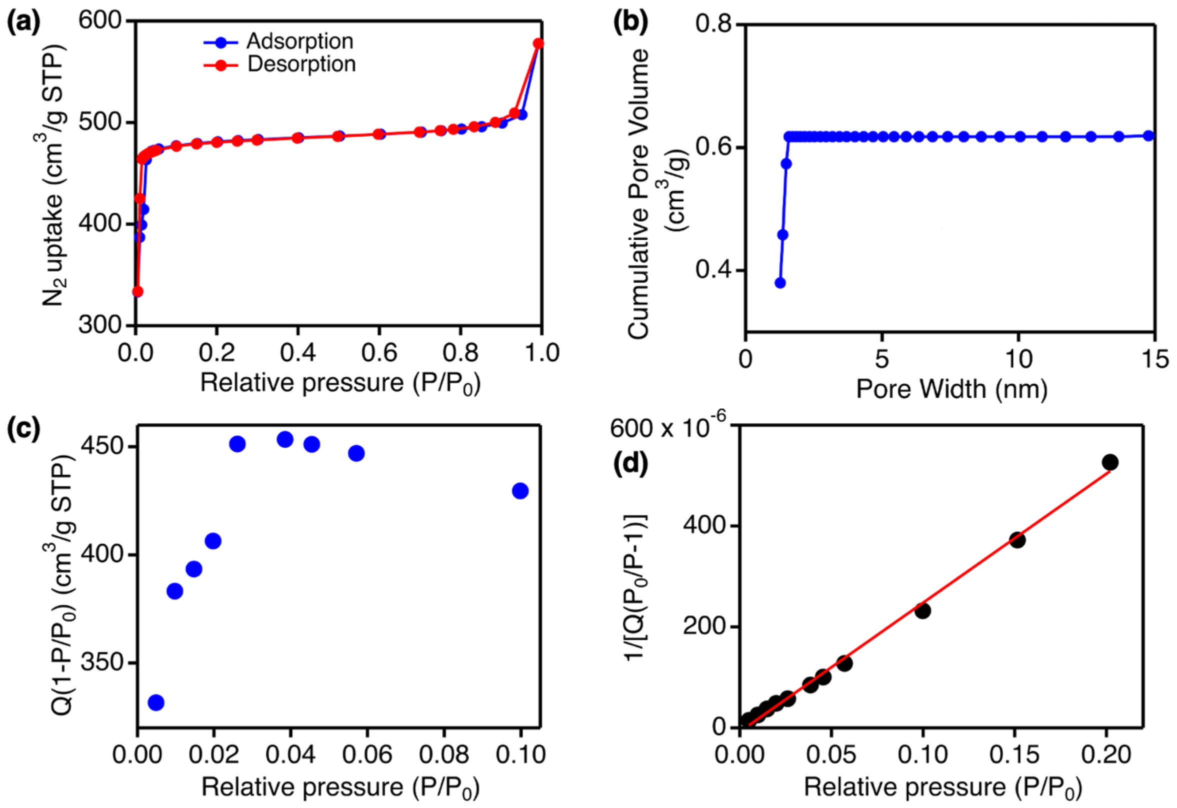


**Figure S5.** (a) Representative nitrogen adsorption and desorption isotherms of the synthesized ZIF-8 nanoparticles. (b) Plot of the cumulative pore volume vs pore width. (c) Plot of $Q \bullet (1 - P/P_0)$ vs. $P/P_0$, where Q is the quantity of $N_2$ in volume. (d) Plot of $1/[Q \bullet (P_0/P-1)]$ vs. $P/P_0$ to determine the BET surface area for ZIF-8. We note that the fits are highly linear across all pressures, with $R^2$ values exceeding 0.99. We quantified the properties of ZIF-8 over two pressure ranges: $P/P_0 = 0.009$ to 0.1, and $P/P_0 = 0.0049$ to 0.035. For both pressure ranges, the pore volume was 0.61 $cm^3/g \pm 0.02$ $cm^3/g$ and the pore width distribution centered on values below 1.5 nm. For data fit over the wider pressure range, the surface area was 1,860 $m^2/g \pm 70$ $m^2/g$; for data fit over the narrower pressure range, the surface area was $2100 \pm 60$ $m^2/g$. These pore volume, pore width, and surface area values are highly consistent with previously-reported values for ZIF-8.[2, 3, 5]

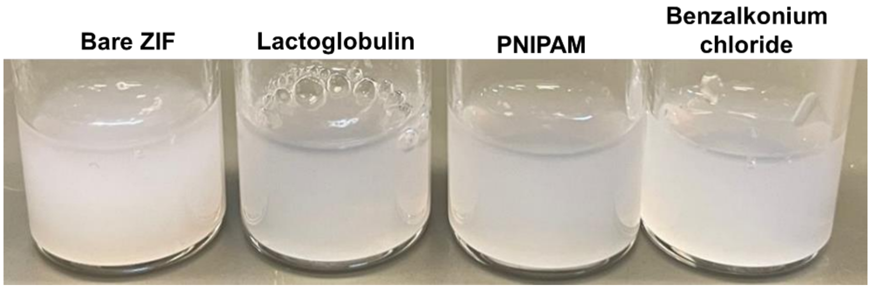


**Figure S6.** Photographs of ZIF-8 dispersions in water with and without surface coatings.

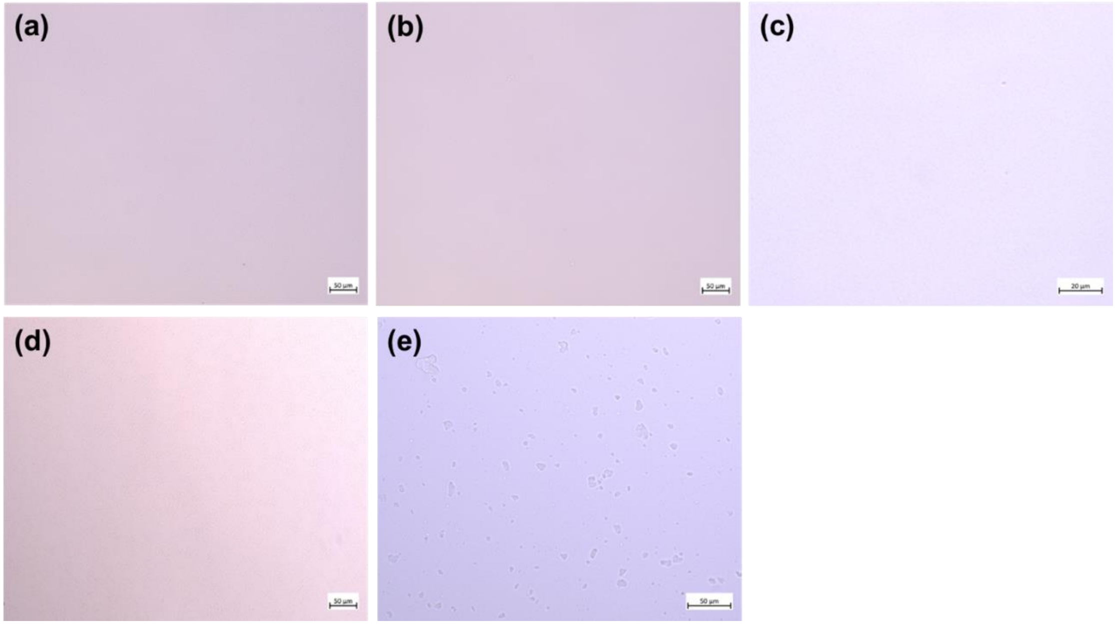


**Figure S7.** Optical micrographs of ZIF-8 (10 mg/mL) in water with (a) no coating (bare ZIF-8), (b) 10 mg/mL PNIPAM, (c) 5 mg/mL lactoglobulin, and (d) 10 mg/mL benzalkonium chloride. (e) Optical micrograph showing that lower concentrations of lactoglobulin (1 mg/mL) lead to aggregation of ZIF-8 particles in water, which we show here as a negative control to compare against the systems that were well dispersed.

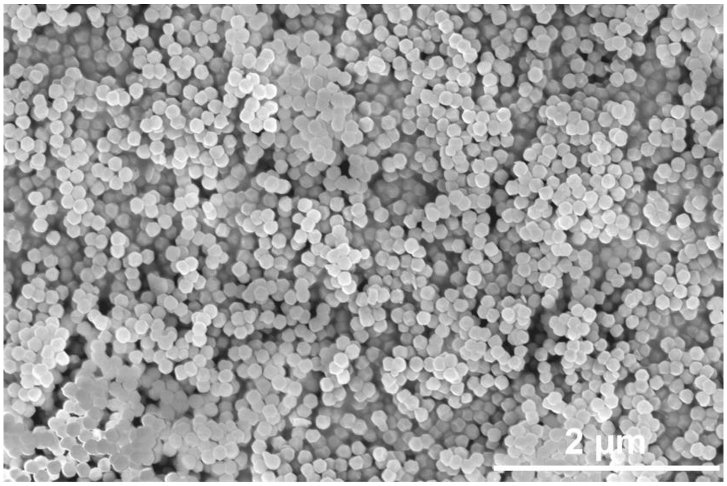


**Figure S8.** Representative SEM image of PEG-filled ZIF-8 particles. No change in particle shape or size was observed for the PEG/ZIF-8 blend relative to as-synthesized, bare ZIF-8 particles.

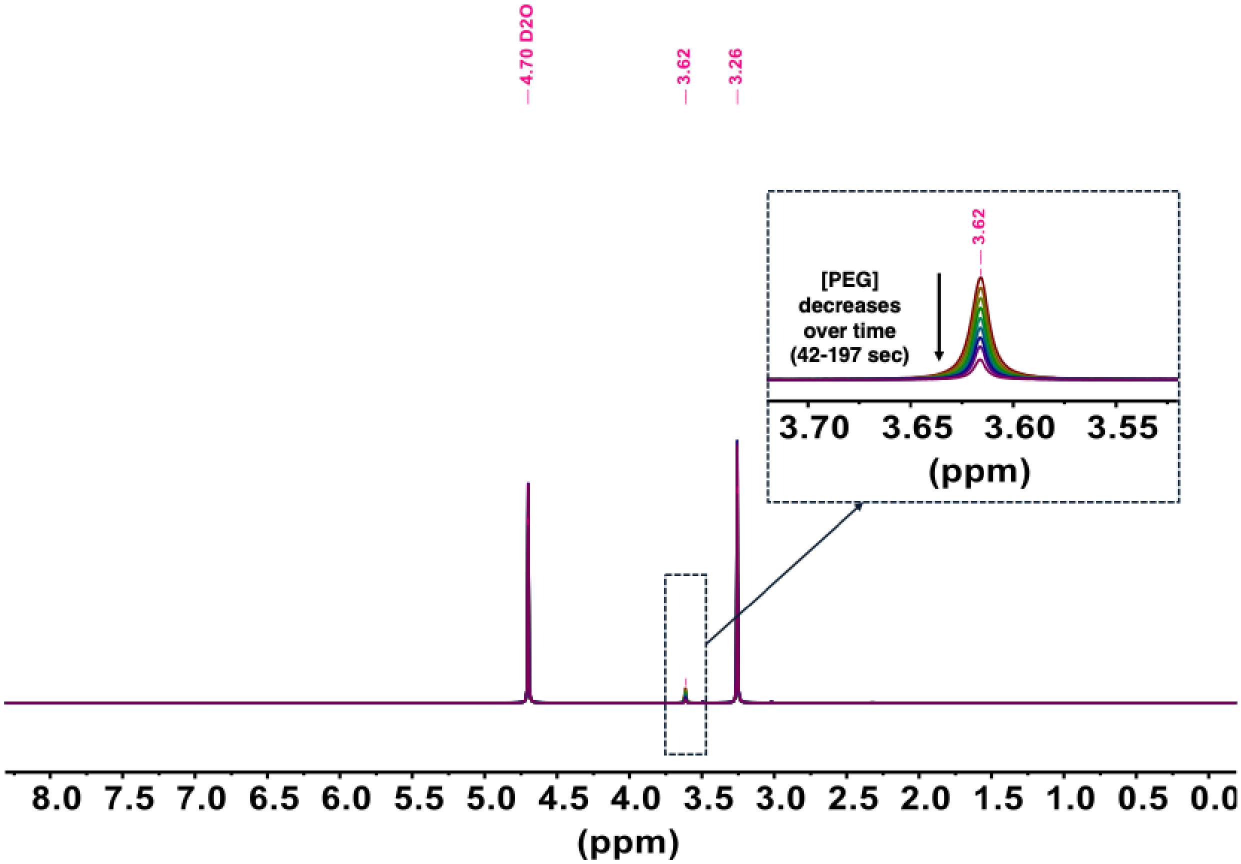


**Figure S9.** Solution-state $^{1}$H-NMR spectra superimposed over time (42-197 sec) for bare ZIF-8 (10 mg/mL) and PEG 35 kDa (3 mg/mL) using the PULCON method.

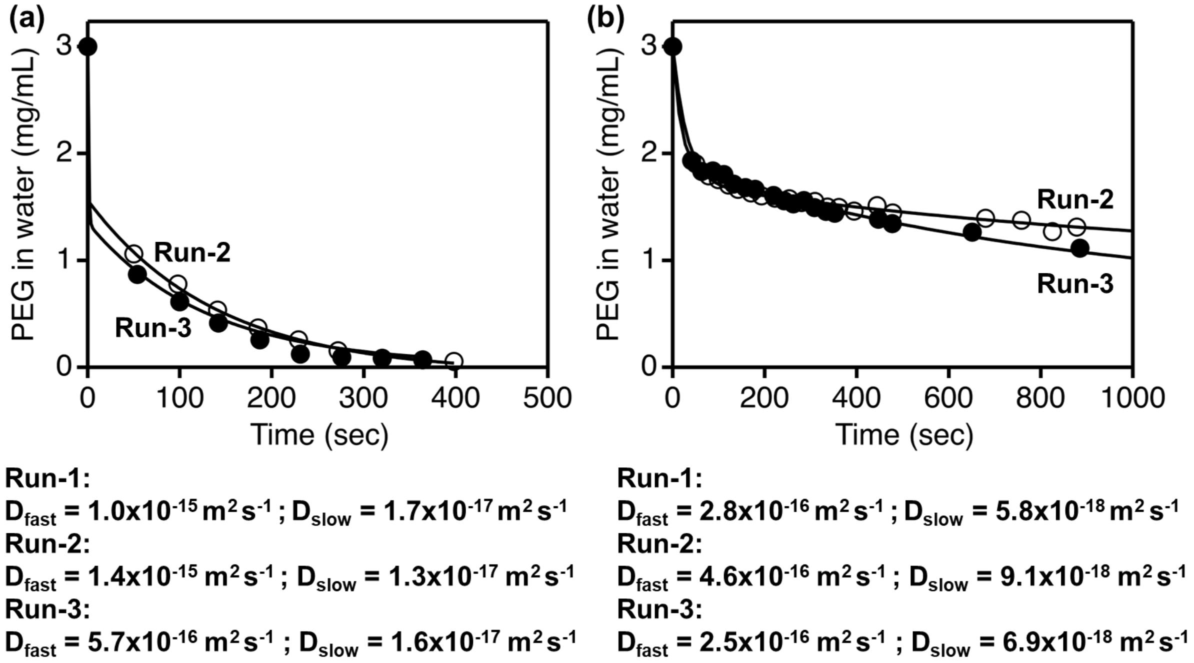


**Figure S10.** Double exponential decay fit of PEG 35 kDa threading into (a) ZIF-8 (240 nm) and (b) ZIF-8 (550 nm) for each experimental run (plots of Run-1 for both (a) and (b) are presented in Figure 2d in the main article). The effective diffusion coefficients of the fast and slow steps for each run are presented below the respective plots.

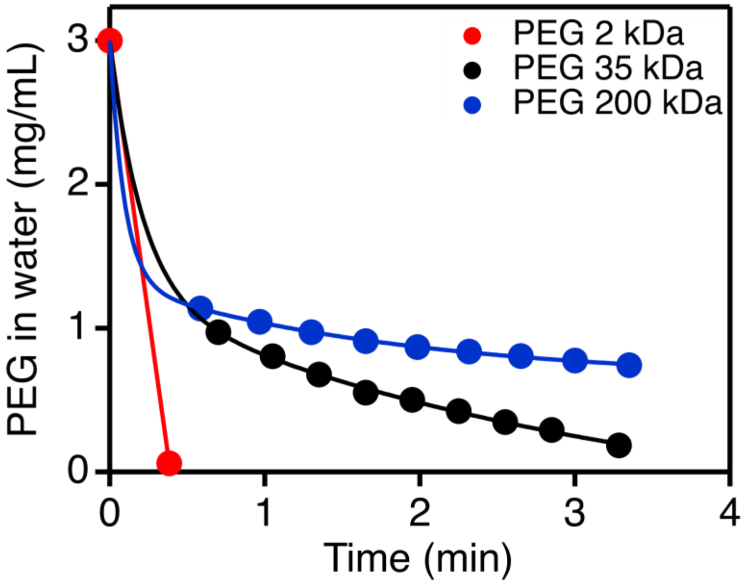


**Figure S11.** Plot showing PEG with three different molecular weights threading from water into ZIF-8 (240 nm) obtained from solution-state NMR. Both PEG 35 kDa ($D_{fast}$ = 1.0x10$^{-15}$ ± 3.5x10$^{-16}$ m$^2$ s$^{-1}$ and $D_{slow}$ = 1.6x10$^{-17}$ ± 1.6x10$^{-18}$ m$^2$ s$^{-1}$) and 200 kDa ($D_{fast}$ = 1.1x10$^{-16}$ ± 2.8x10$^{-17}$ m$^2$ s$^{-1}$ and $D_{slow}$ = 3.4x10$^{-18}$ ± 1.3x10$^{-18}$ m$^2$ s$^{-1}$) fit to the double exponential decay, and the diffusion of PEG 200 kDa is slowed down by an order of magnitude as compared to PEG 35 kDa. PEG 2 kDa threads too fast into bare ZIF-8, so the effective diffusion coefficient cannot be quantified.

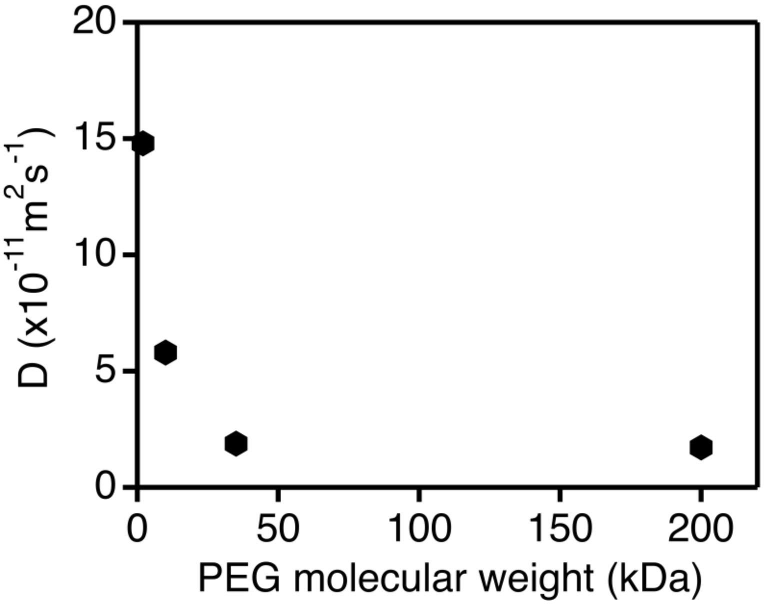


**Figure S12.** Self-diffusion coefficients of pure PEG as a function of molecular weight measured in water with a pulsed field gradient (PFG) spin-echo pulse sequence. PEG concentration was fixed at 3 mg/mL for each run.

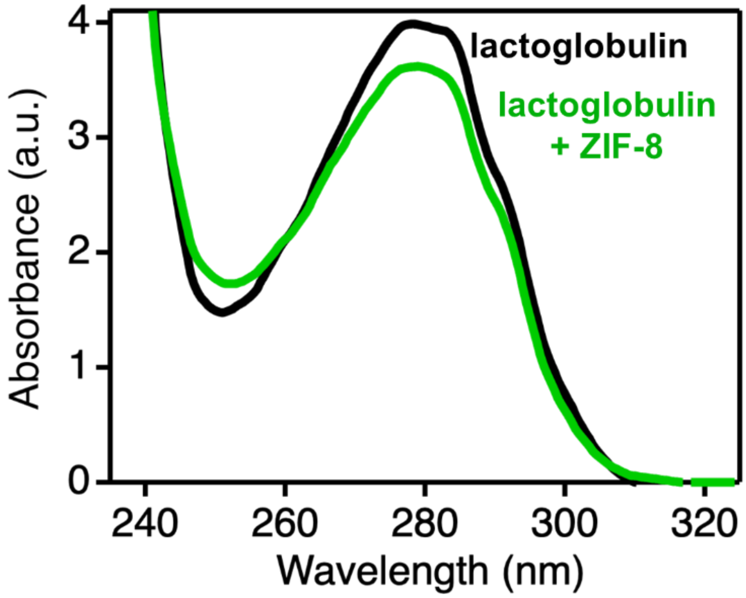


**Figure S13.** UV-visible spectra for pure lactoglobulin in water (5 mg/mL) (black) and the supernatant of dispersions made of ZIF-8 (10 mg/mL) + lactoglobulin (5 mg/mL) in water (green). The difference from pure lactoglobulin to lactoglobulin in the supernatant of ZIF-8 dispersions was taken to be the amount of lactoglobulin adsorbed on the ZIF-8 surface.

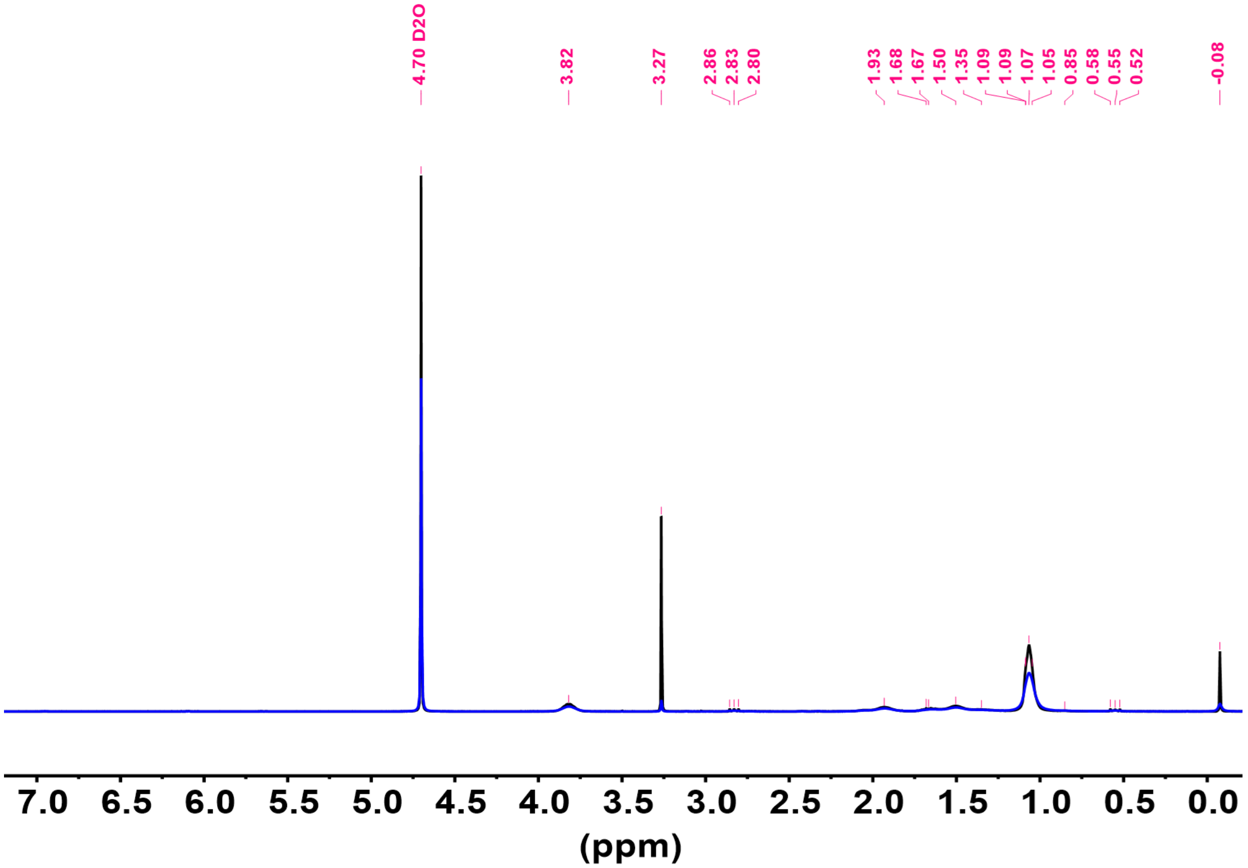


**Figure S14.** $^1$H-NMR spectra for pure PNIPAM in $D_2O$ (10 mg/mL) (black) and ZIF-8:PNIPAM (10:10 mg/mL) dispersion in $D_2O$ (blue). The difference in concentration between pure PNIPAM and the PNIPAM in the supernatant of ZIF-8+PNIPAM was taken to be the amount of PNIPAM adsorbed to the external surface of ZIF-8.

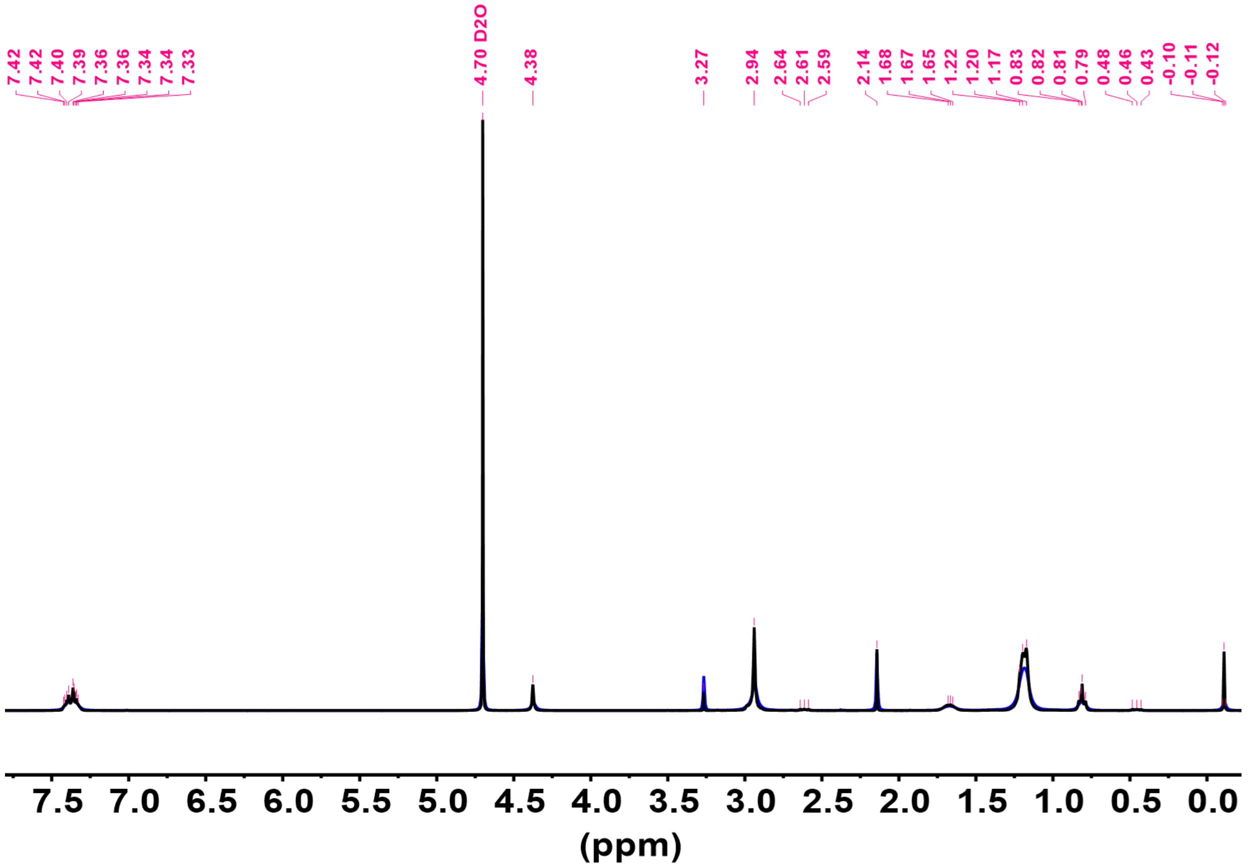


**Figure S15.** $^{1}$H-NMR spectra for pure benzalkonium chloride in $D_2O$ (10 mg/mL) (black) and ZIF-8:benzalkonium chloride (10:10 mg/mL) dispersion in $D_2O$ (blue). The difference in concentration between pure benzalkonium chloride and the benzalkonium chloride in the supernatant of ZIF-8+benzalkonium chloride was taken to be the amount of benzalkonium chloride adsorbed to the external surface of ZIF-8.

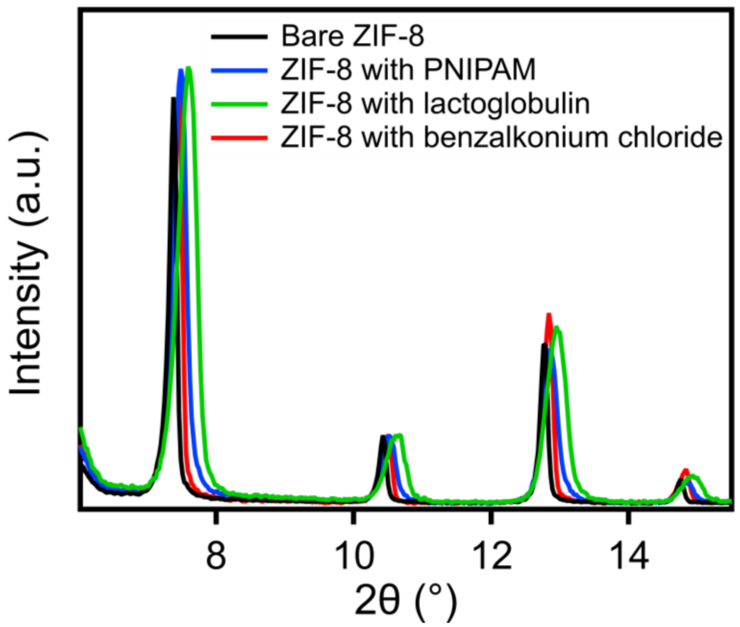


**Figure S16.** PXRD patterns of ZIF-8 particles coated with different molecules, showing negligible differences in the relative intensity of the 110 reflection (which suggests that the molecules remain localized to the external ZIF-8 surface and preserve empty micropores).

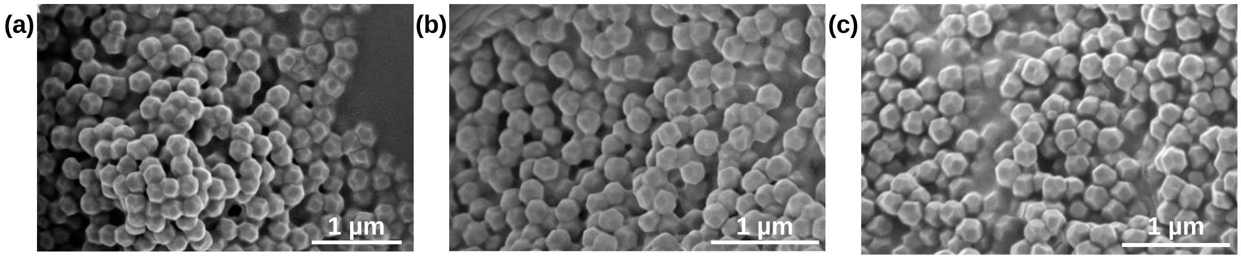


**Figure S17.** Representative SEM images of ZIF-8 particles coated with (a) PNIPAM, (b) lactoglobulin, and (c) benzalkonium chloride. No change in particle shape or size was observed for any of the coatings relative to that of as-synthesized, bare ZIF-8 particles.

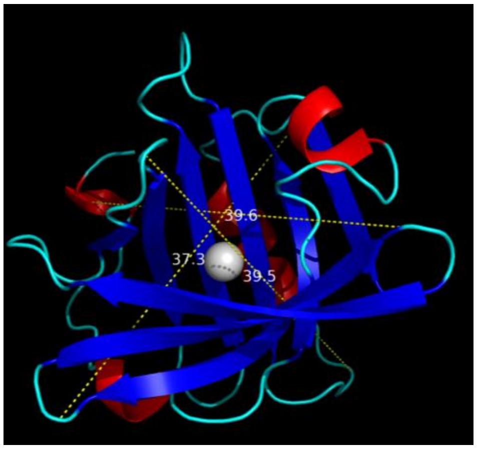


**Figure S18.** Pymol image of lactoglobulin (PDB code: 1b8e). The radius of lactoglobulin was calculated from PyMol by taking an average of three measured diameters (image shows the values in Å). This analysis was used to estimate the footprint of lactoglobulin (11.34 nm$^2$), assuming a circular projection onto the ZIF-8 surface.

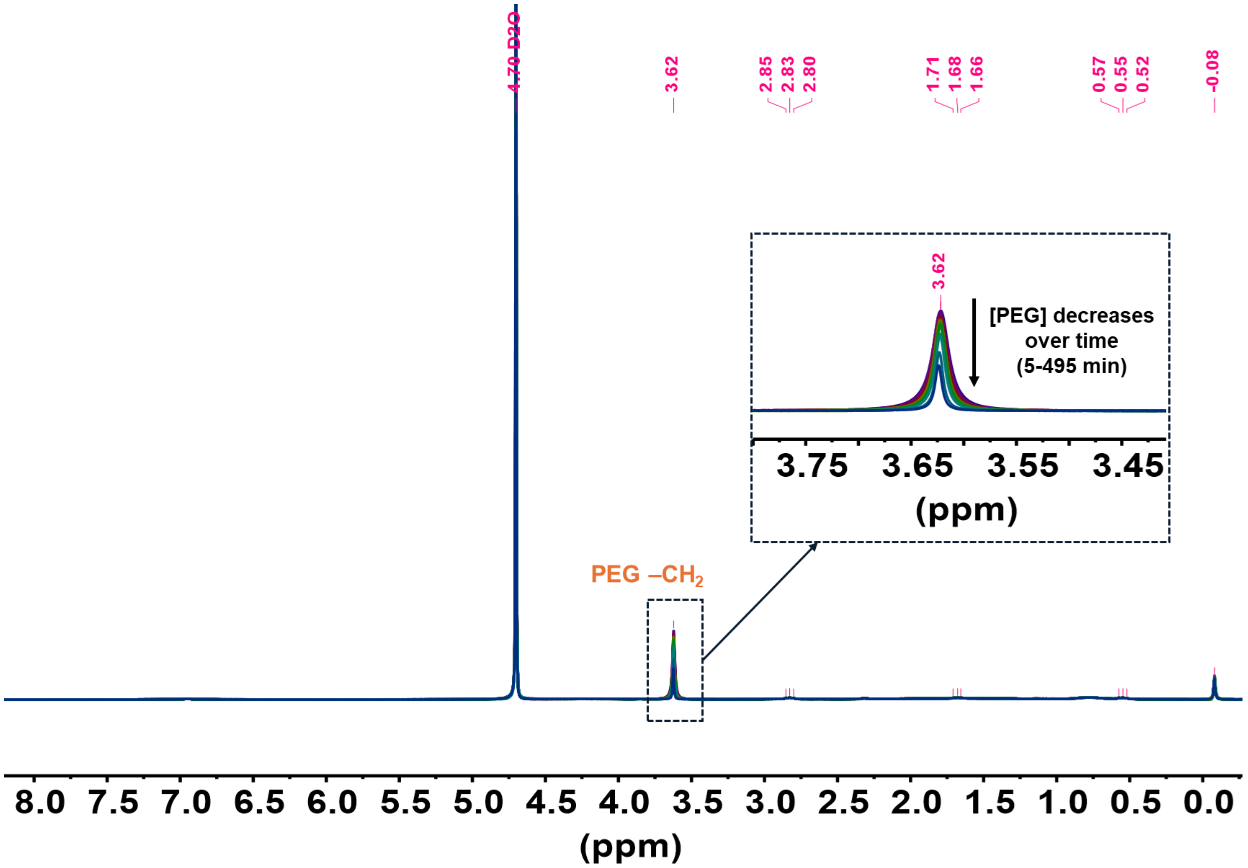


**Figure S19.** Solution-state $^1$H-NMR spectra superimposed over time (5-495 min) for ZIF-8 (10 mg/mL) and PEG 35 kDa (3 mg/mL), where the ZIF-8 particles are coated with lactoglobulin (5 mg/mL).

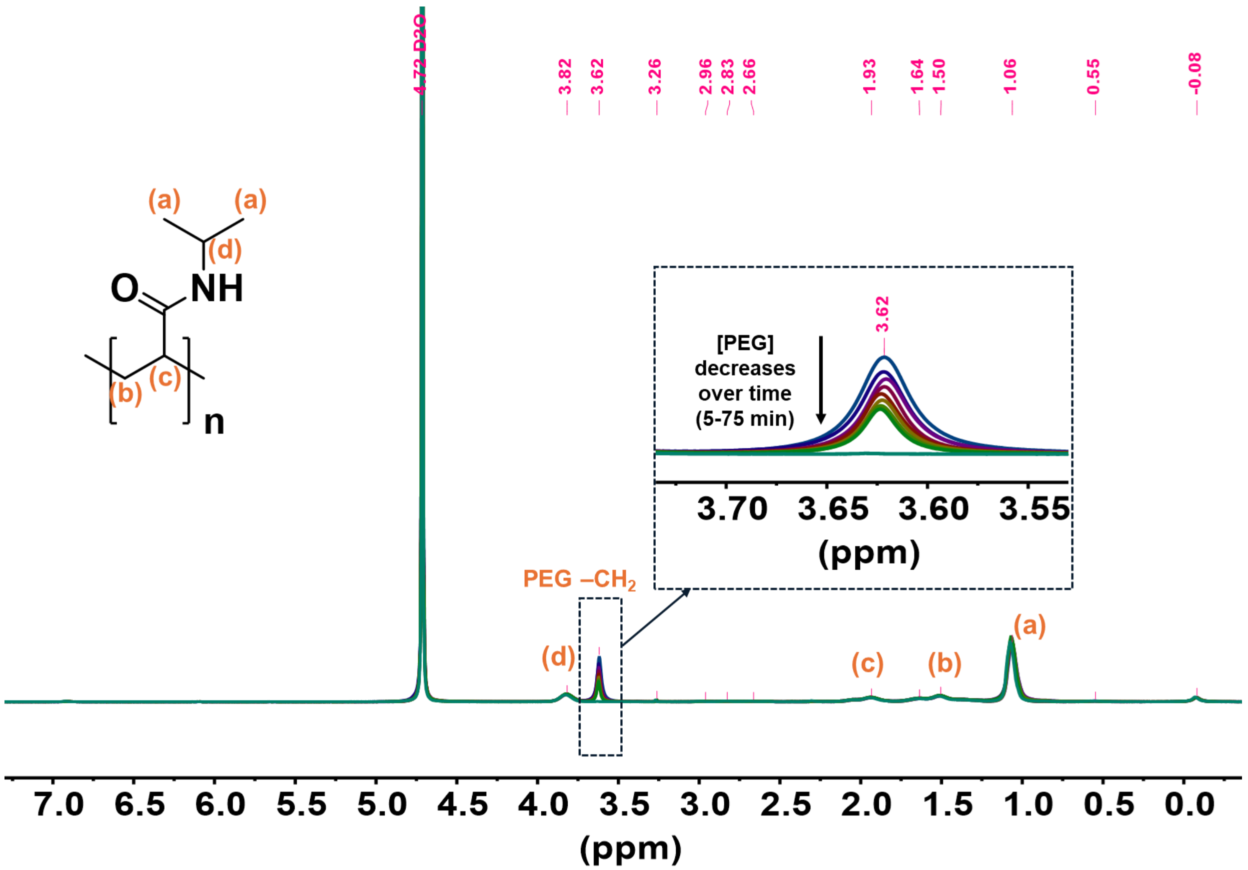


**Figure S20.** Solution-state $^1$H-NMR spectra superimposed over time (5-75 min, and ~22 h for the spectrum showing no PEG signal) for ZIF-8 (10 mg/mL) and PEG 35 kDa (3 mg/mL), where the ZIF-8 particles are coated with PNIPAM (10 mg/mL).

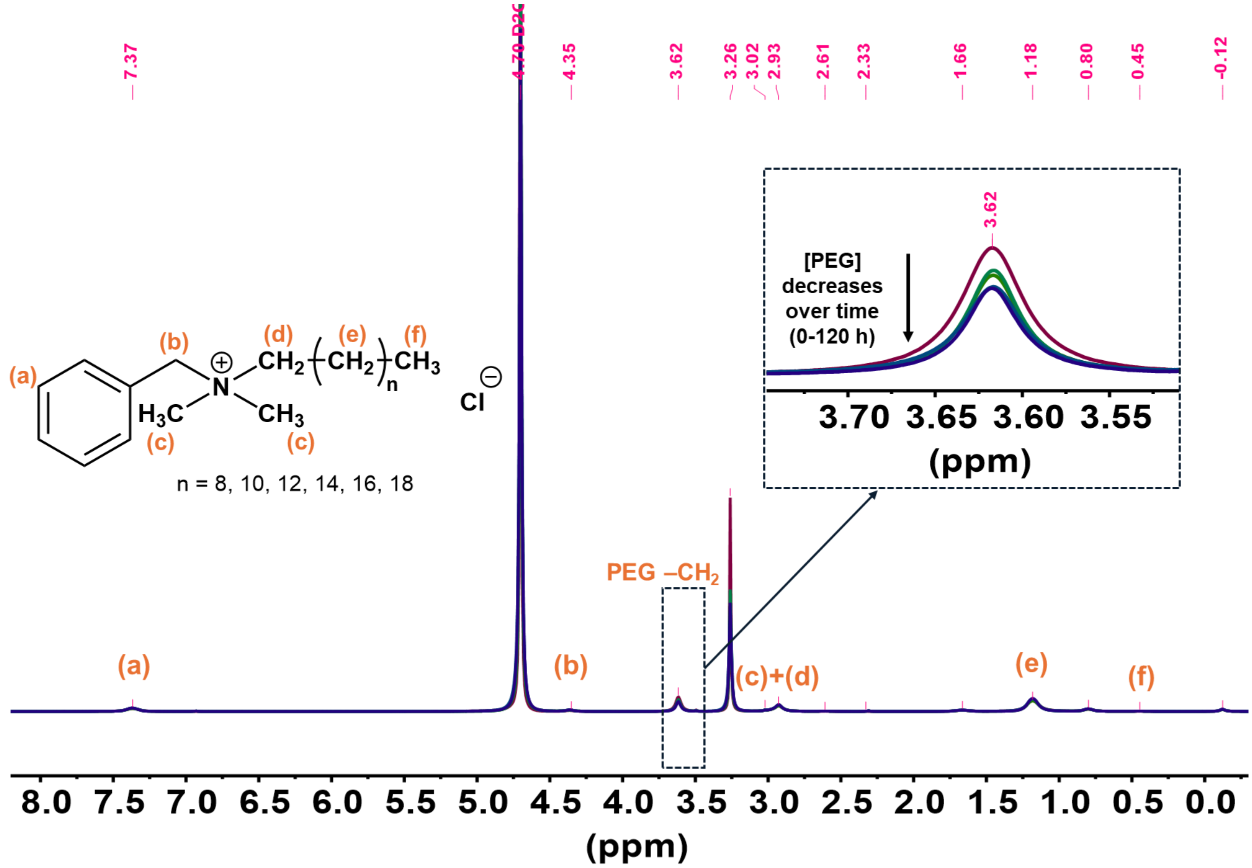


**Figure S21.** Solution-state $^{1}$H-NMR spectra superimposed over time (0-120 h) for ZIF-8 (10 mg/mL) and PEG 35 kDa (3 mg/mL), where the ZIF-8 particles are coated with benzalkonium chloride (10 mg/mL).

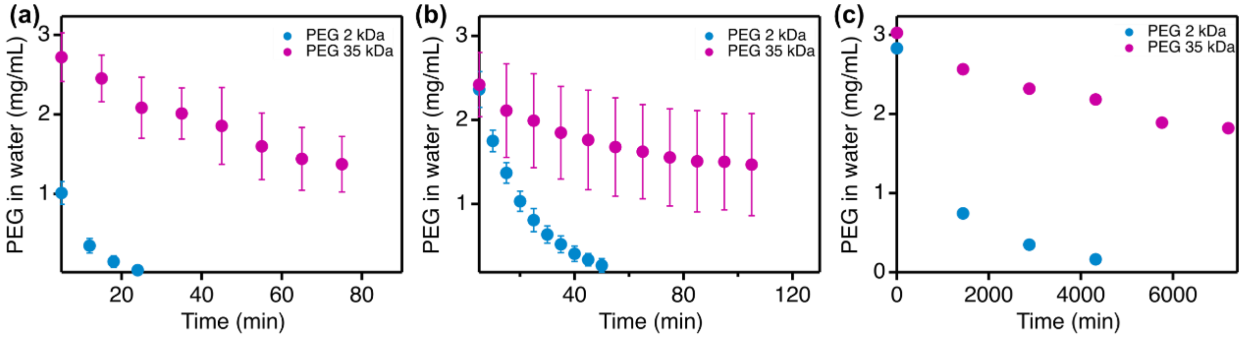


**Figure S22.** Threading of PEGs (3 mg/mL) into ZIF-8 (10 mg/mL) coated with: (a) PNIPAM (10 mg/mL), (b) lactoglobulin (5 mg/mL), and (c) benzalkonium chloride (10 mg/mL).

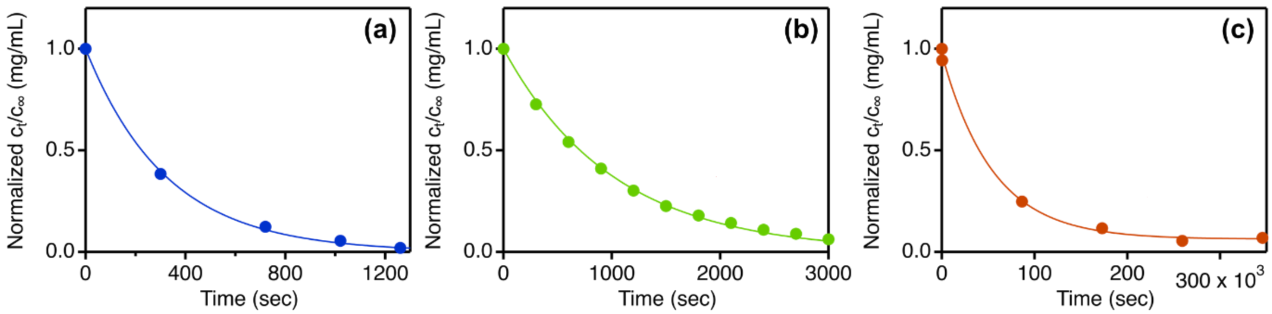


**Figure S23.** Representative plots and fits for threading of PEG 2 kDa into surface-functionalized ZIF-8 with (a) PNIPAM, (b) lactoglobulin, and (c) benzalkonium chloride. The plots show the fitting curves from equation S2 mentioned in Section 6 above and analyzed in R Studio. The same fits were used to quantify effective diffusivities for PEG 35 kDa.

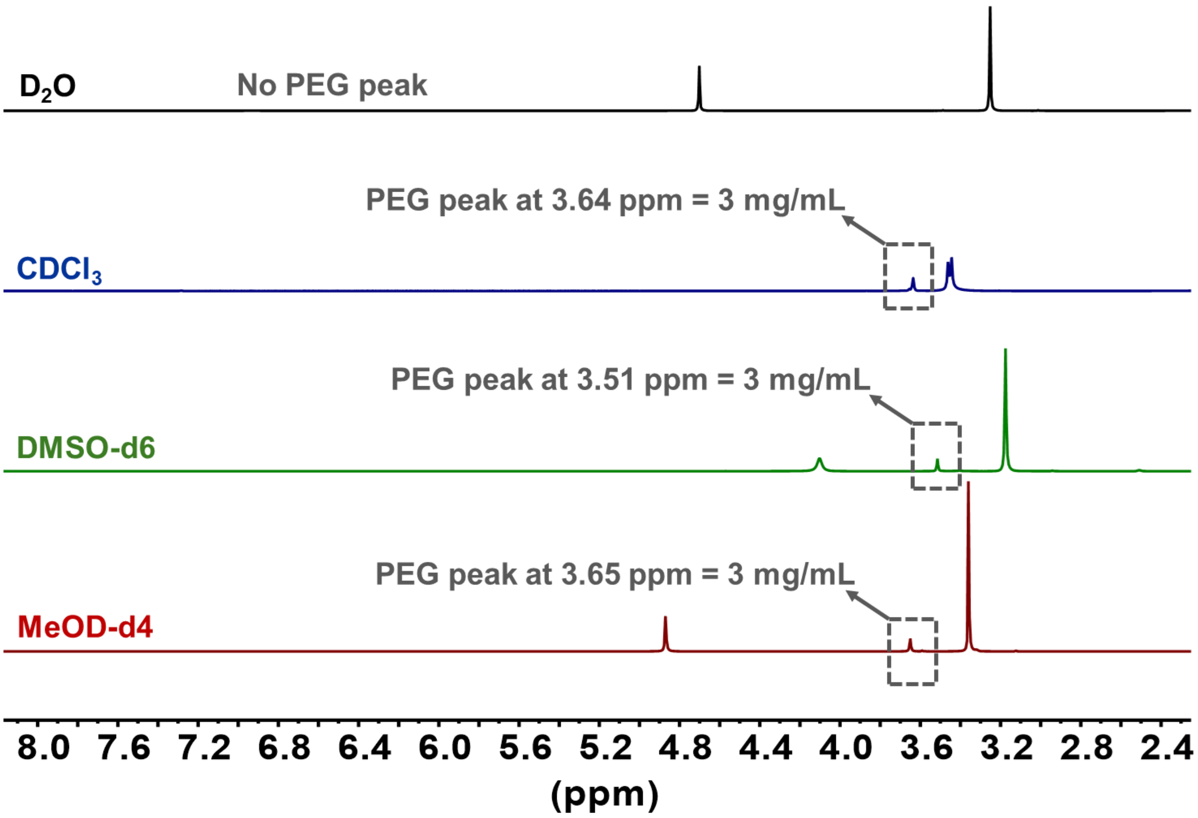


**Figure S24.** Stacked solution-state $^1$H-NMR spectra of ZIF-8 (10 mg/mL) with PEG 2 kDa (3 mg/mL) in different deuterated solvents. The concentrations of PEG are calculated from the absolute integral of the PEG signal in different solvents.

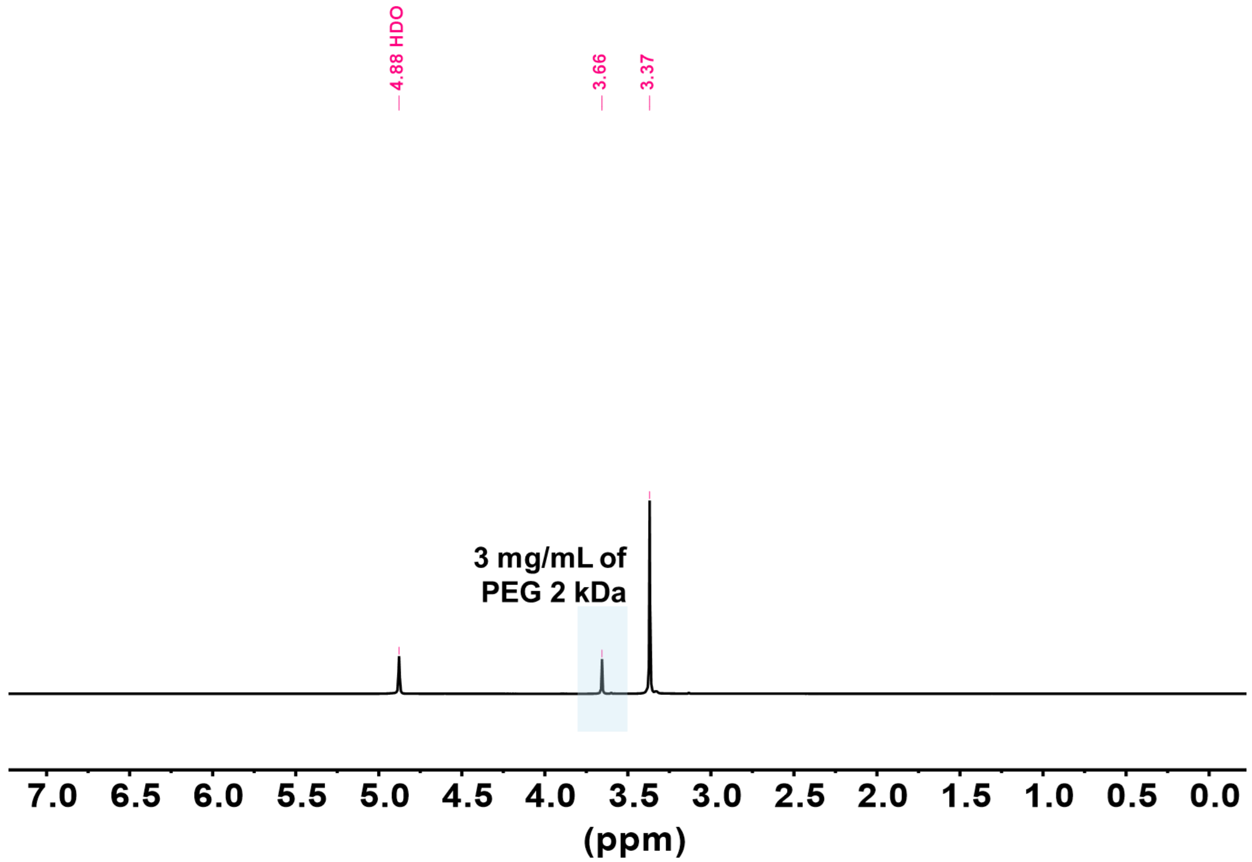


**Figure S25.** Solution-state $^1$H-NMR spectra of the supernatant of ZIF-8 (10 mg/mL) + PEG 2 kDa (3 mg/mL) in MeOD, showing that PEG does not thread into the ZIF-8 pores and remains outside dissolved in solution.

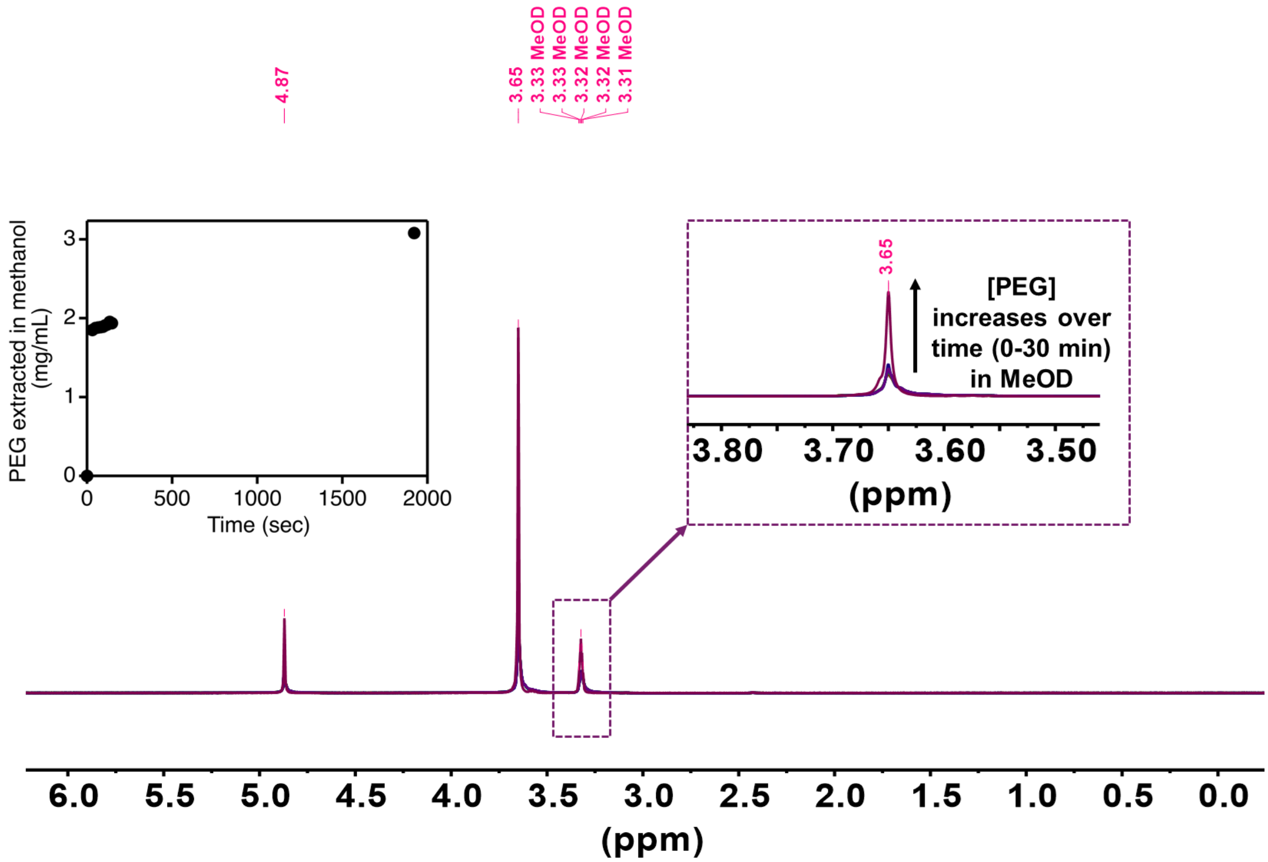


**Figure S26.** Solution-state $^1$H-NMR spectra over time of PEG-filled ZIF-8 particles redispersed in methanol. Bare ZIF-8 particles (10 mg/mL) were first blended with PEG (2kDa) (3 mg/mL) in water for 30 min to allow all PEG chains to fill the pores. The particles were then collected, dried, and redispersed in deuterated methanol at 10 mg/mL ZIF-8. The concentration of PEG in methanol rapidly increases, showing that PEG de-threads completely (3 mg/mL) from the ZIF-8 pores into the solvent within 30 min.

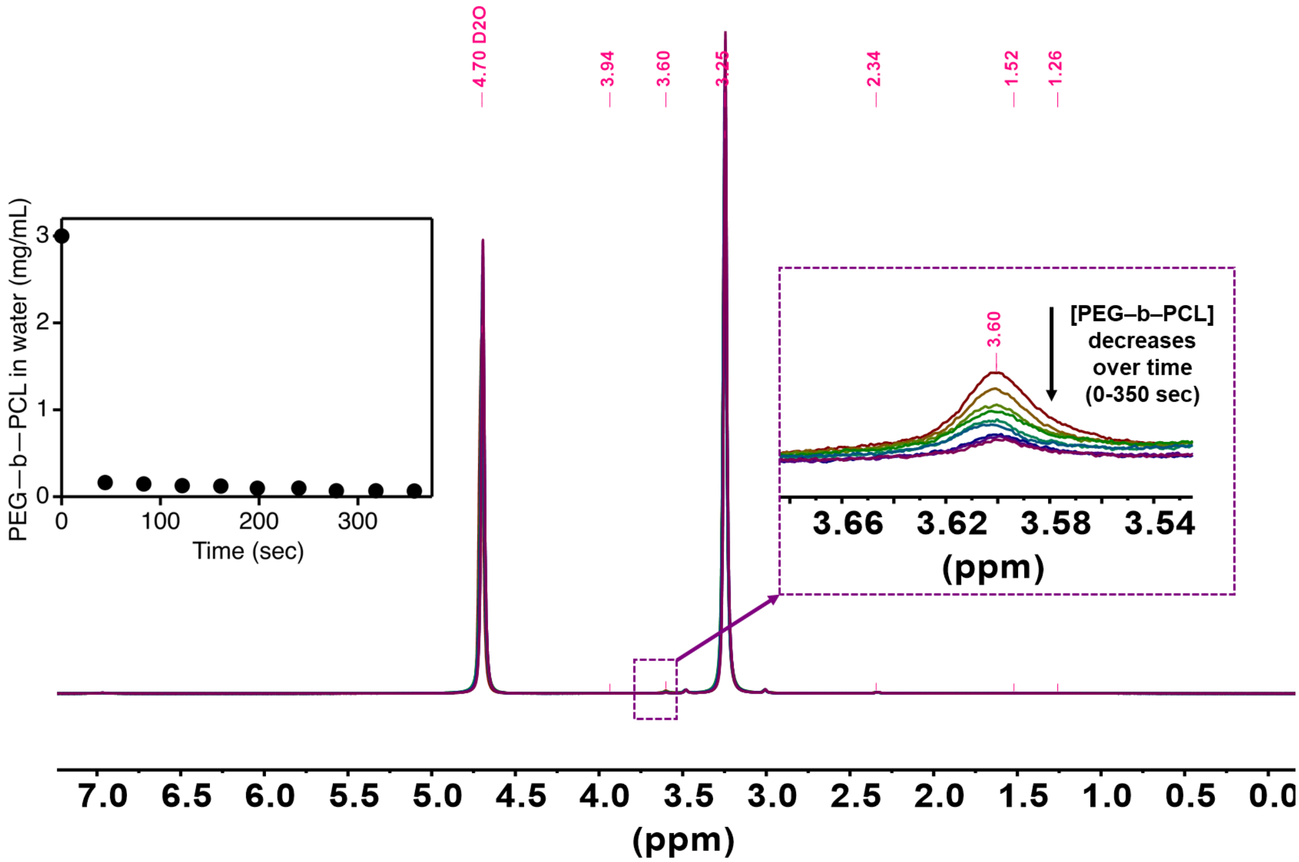


**Figure S27.** Solution-state $^{1}$H-NMR spectra superimposed over time (0-350 sec) for ZIF-8 (10 mg/mL) and poly(ethylene glycol–b–caprolactone) (PEG–b–PCL) 2–b–2.6 kDa (3 mg/mL) recorded in $D_2O$. The graph on the left side shows that PEG–b–PCL completely threads into ZIF-8 within ~350 sec (and >90% of the chains are inside ZIF-8 within 50 sec).

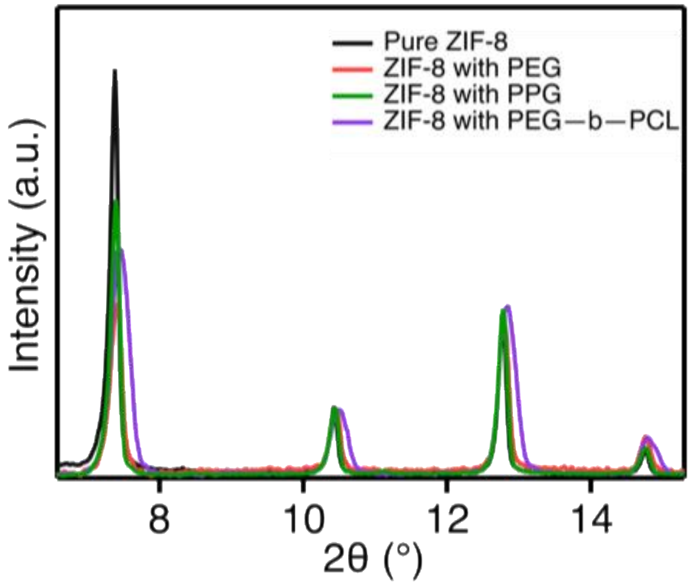


**Figure S28.** PXRD patterns of ZIF-8 particles with PPG 2 kDa and block polymer PEG–b–PCL 2–b–2.6 kDa.

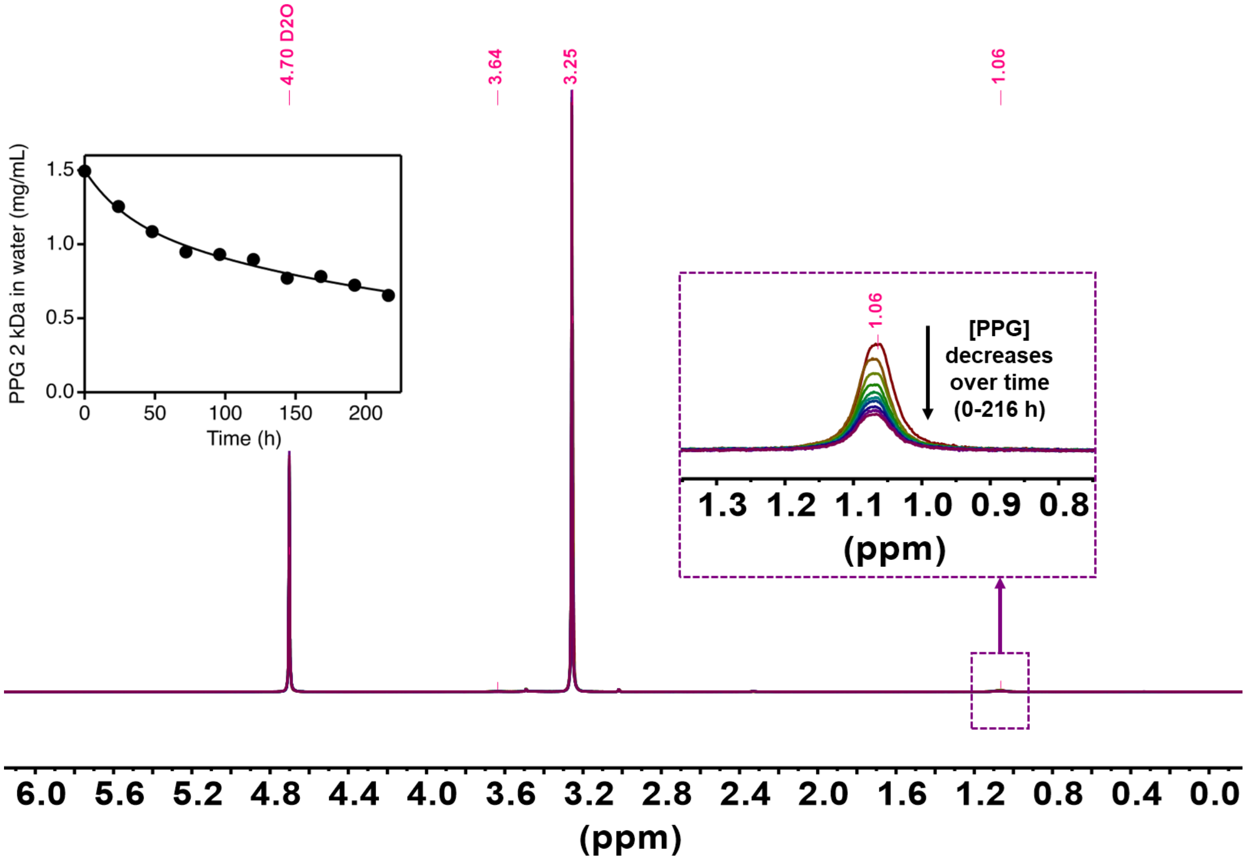


**Figure S29.** Solution-state $^{1}$H-NMR spectra superimposed over time (0-216 h) for ZIF-8 (5 mg/mL) and polypropylene glycol (PPG) 2 kDa (1.5 mg/mL) recorded in $D_2O$. The graph on the left side shows that PPG threading into ZIF-8 follows a double exponential decay.

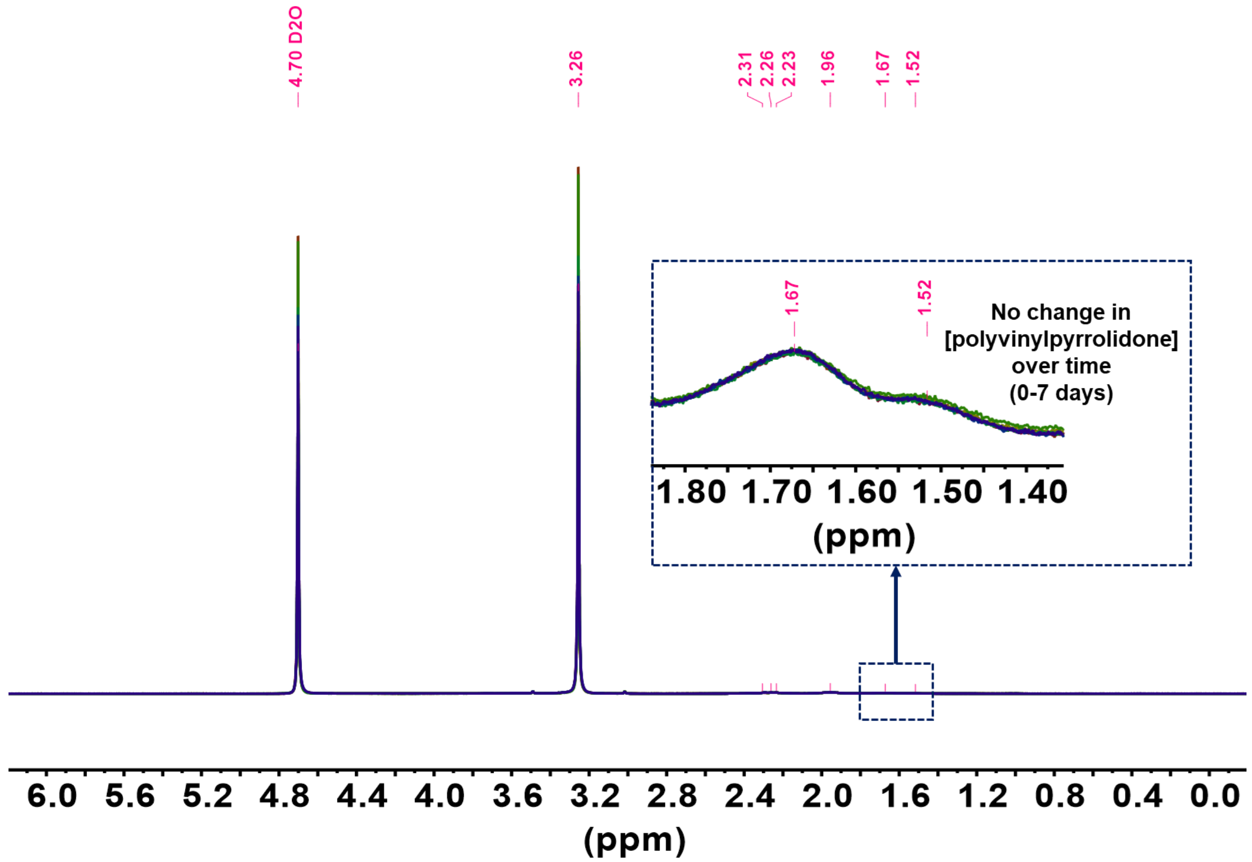


**Figure S30.** Solution-state $^{1}$H-NMR spectra superimposed over time (0-7 days) for ZIF-8 (10 mg/mL) and polyvinylpyrrolidone (PVP) 10 kDa (3 mg/mL).

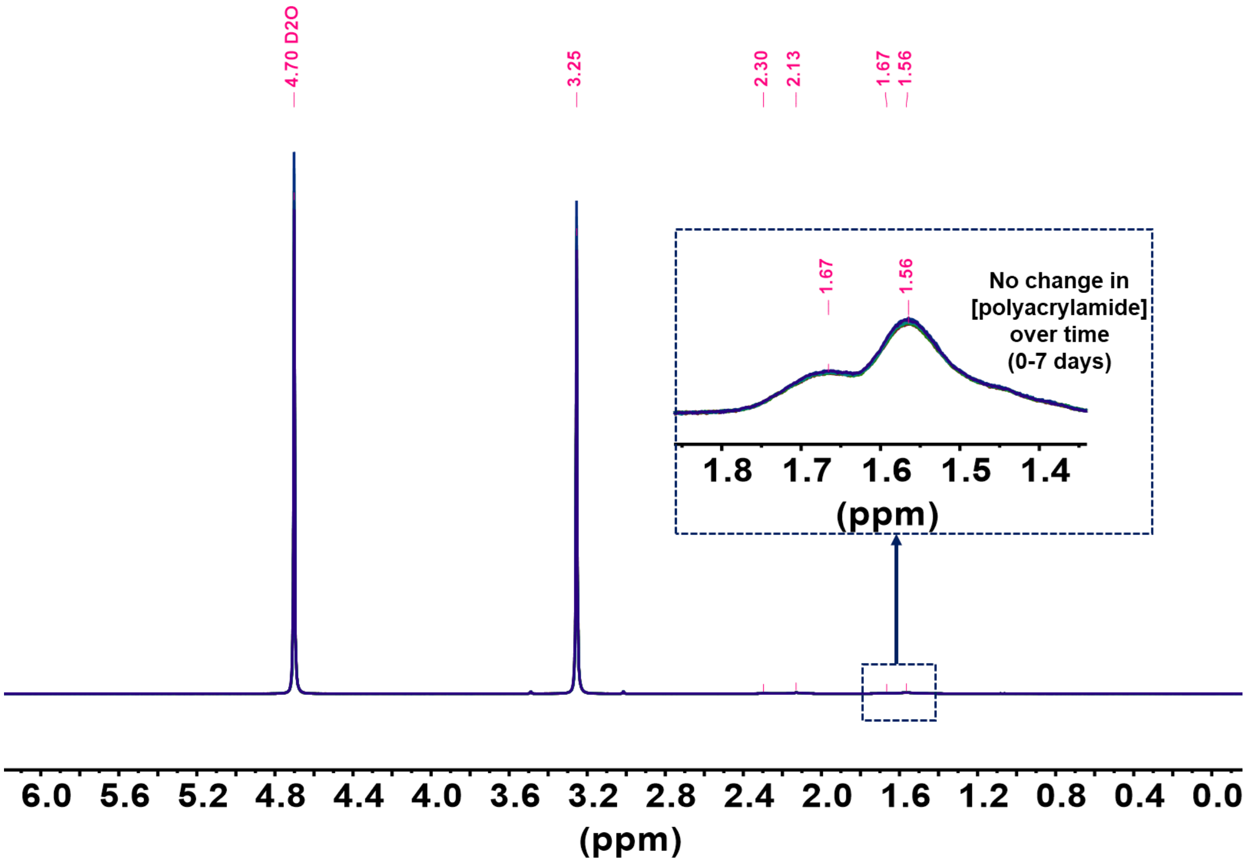


**Figure S31.** Solution-state $^{1}$H-NMR spectra superimposed over time (0-7 days) for ZIF-8 (10 mg/mL) and polyacrylamide 10 kDa (3 mg/mL).

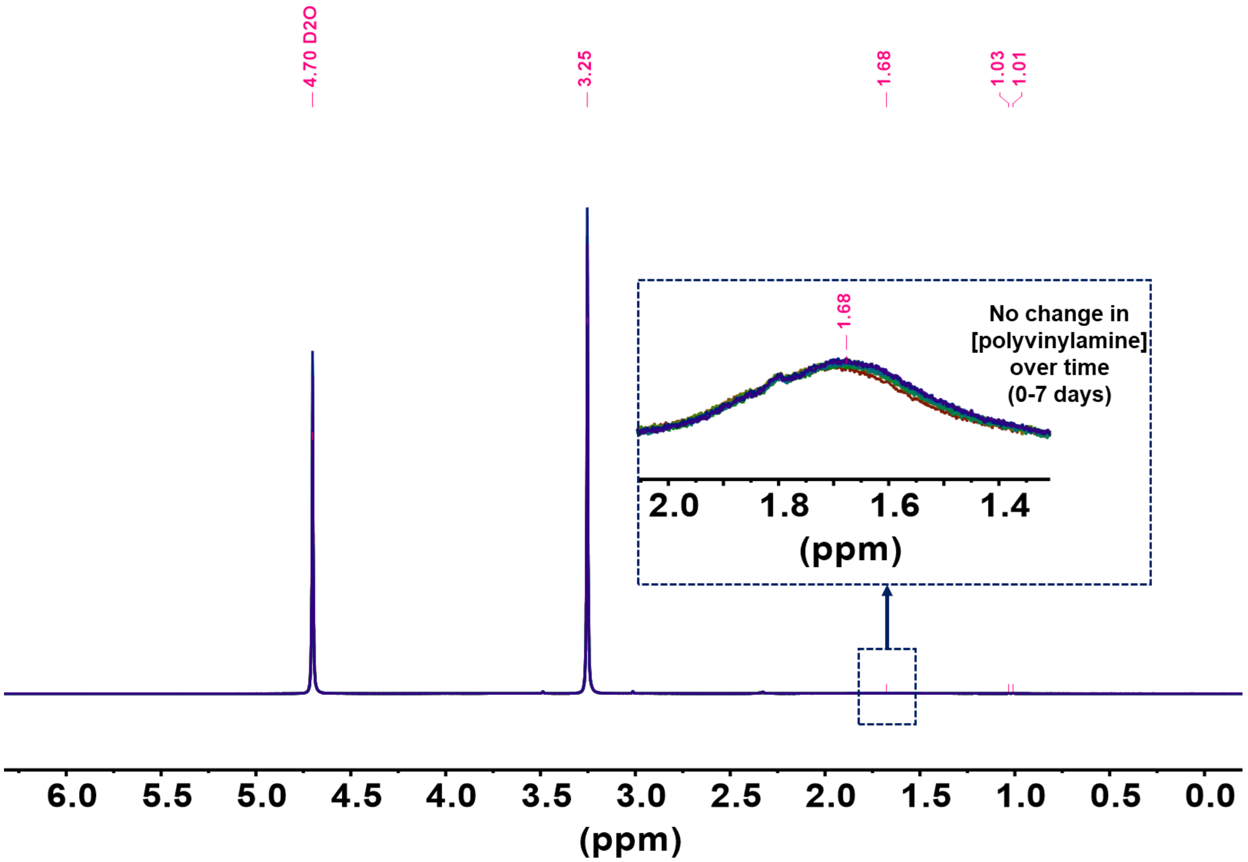


**Figure S32.** Solution-state $^{1}H$-NMR spectra superimposed over time (0-7 days) for ZIF-8 (10 mg/mL) and polyvinylamine 9 kDa (3 mg/mL).

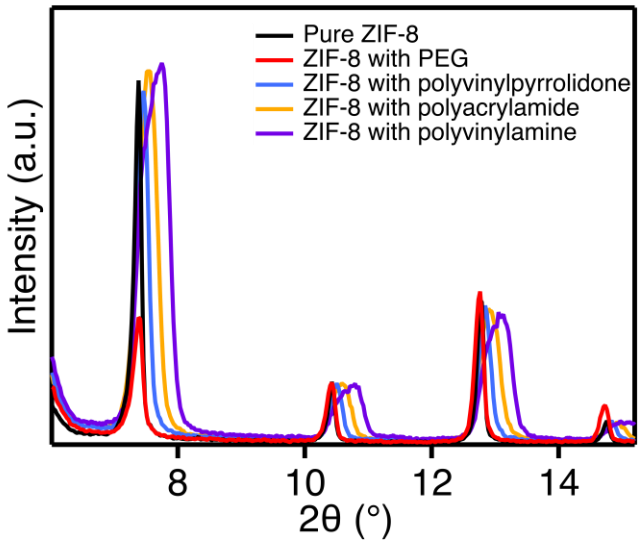


**Figure S33.** PXRD patterns of ZIF-8 particles with polyvinylpyrrolidone (PVP), polyacrylamide, and polyvinylamine showing no intensity differences (these polymers do not fill up the pores).

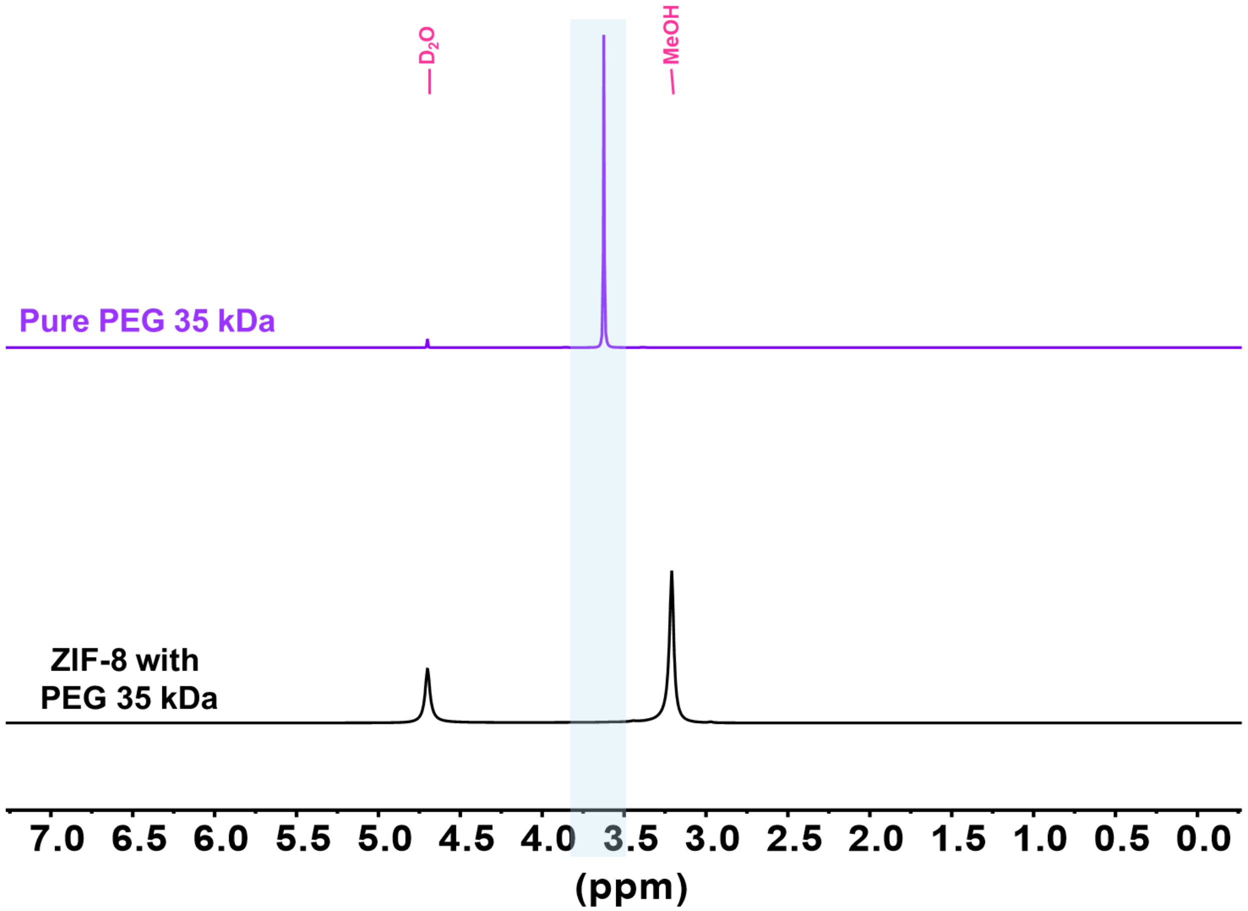


**Figure S34.** Solution-state $^1$H-NMR spectra for pure PEG 35 kDa (30 mg/mL) and ZIF-8 (100 mg/mL) + PEG 35 kDa (30 mg/mL) recorded after 10 min of mixing in $D_2O$. The PEG peak is invisible in the ZIF-8/PEG blend, showing that PEG threading inside the ZIF-8 pores can be measured at relatively high concentrations.

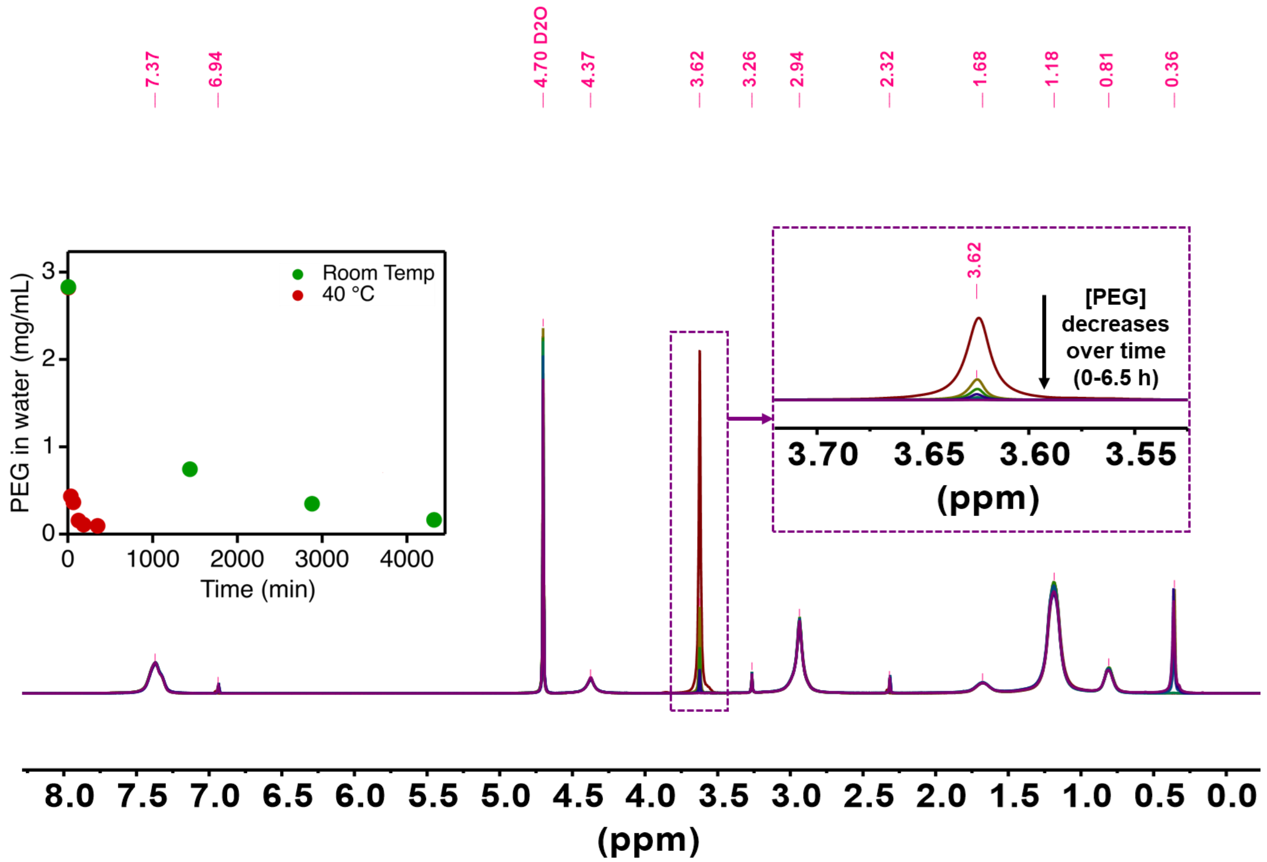


**Figure S35.** Solution-state $^1$H-NMR spectra superimposed over time (0-6.5 h) for ZIF-8 (10 mg/mL) and PEG 2 kDa (3 mg/mL) at 40 °C, where the ZIF-8 particles are coated with benzalkonium chloride (10 mg/mL). The comparison of PEG threading at different temperatures on the left side shows that the threading rate increases substantially with increasing temperature.

**References:**


1. DelRe, C.; Hong, H.; Wenny, M. B.; Erdosy, D. P.; Cho, J.; Lee, B.; Mason, J. A., Design Principles for Using Amphiphilic Polymers To Create Microporous Water. *J. Am. Chem. Soc.* **2023**, *145* (36), 19982-19988.
2. Park, K. S.; Ni, Z.; Cote, A. P.; Choi, J. Y.; Huang, R. D.; Uribe-Romo, F. J.; Chae, H. K.; O'Keeffe, M.; Yaghi, O. M., Exceptional chemical and thermal stability of zeolitic imidazolate frameworks. *P Natl Acad Sci USA* **2006,** *103* (27), 10186-10191.
3. Erdosy, D.P.; Wenny, M. B.; Cho, J. et al. Microporous water with high gas solubilities. *Nature* **2022**, *608*, 712-718.
4. Walton, K. S.; Snurr, R. Q., Applicability of the BET method for determining surface areas of microporous metal-organic frameworks. *J. Am. Chem. Soc.* **2007**, *129*, 8552-8556.
5. Agrawal, M.; Han, R.; Herath, D.; Sholl, D.S., Does repeat synthesis in materials chemistry obey a power law? *Proc Natl Acad Sci USA* **2020**, *117 (2)* 877-882.
6. Roquerol, J.; Llewellyn, P.; Rouquerol, F., Is the bet equation applicable to microporous adsorbents? *Studies in Surface Science and Catalysis* **2007** *160*, 49-56.

7. Mak, J. Y. W., Determination of Sample Concentrations by PULCON NMR Spectroscopy. *Aust J. Chem.* **2022**, *75* (2), 160-164.
8. Wider, G.; Dreier, L., Measuring Protein Concentrations by NMR Spectroscopy. *J. Am. Chem. Soc.* **2006**, *128* (8), 2571-2576.
9. Ueda, T.; Yamatani, T.; Okumura, M., Dynamic Gate Opening of ZIF-8 for Bulky Molecule Adsorption as Studied by Vapor Adsorption Measurements and Computational Approach. *J. Phys. Chem. C* **2019**, *123* (45), 27542-27553.
